# Supplementary material for: Graphical integrity issues in open access publications: Detection and patterns of proportional ink violations
Source: PLoS Comput Biol. 2021 Dec 13;17(12):e1009650. doi: 10.1371/journal.pcbi.1009650 (PMC8700024; doi:10.1371/journal.pcbi.1009650)
Supplement: S1 Text — (DOCX) [file pcbi.1009650.s008.docx]

**Statistical analysis for annotation pipeline**

In this subsection, we present statistical analysis on proportional ink violations among bar charts in annotation pipeline. Because we have binary labels (proportional ink violation or other) for bar charts in our annotation pipeline, we did not conduct the same statistical analysis to examine the graphical integrity differences across author seniority, impact of journal, research field, affiliation country.

1. **Graphical integrity differences by seniority**

We conducted a Welch two sample t-test to compare if bar charts with proportional ink violation are from lower author seniority compared to bar charts without proportional ink violation. Our result show that there is no statistical significant difference between the h-index of the first author of the publication of bar charts with proportional ink violation and the h-index of the first author of the publication of bar charts without proportional ink violation (Welch’s t-test, t(263.47) = 0.42, *p*-value = 0.67).  Our result also show that there is no statistical significant difference between the h-index of the last author of the publication of bar charts with proportional ink violation and the h-index of the last author of the publication of bar charts without proportional ink violation (Welch’s t-test, t(294.9) = 0.15, *p*-value = 0.88).

1. **Graphical integrity across the impact of journals**

We conducted a Welch two sample t-test to compare if journals with a higher likelihood (the average likelihood across publications greater than 0.5) of proportional ink violation are from lower ranked journals compared to journals with a lower likelihood (the average likelihood across publications less than 0.5) of proportional ink violation. Our result show that bar charts with proportional ink violation are from slightly lower ranked journals (Welch’s t-test, t(24.00) = -0.39 , *p*-value = 0.70).

1. **Graphical integrity across research fields**

We conducted a Pearson’s Chi-squared test to compare if bar charts with proportional ink violation are more likely from certain research fields compared to bar charts without proportional ink violation. Our result show that some research fields are more likely to have bar charts with proportional ink violation (Pearson’s Chi-squared test (based on 2000 replicates), X-squared (NA) =50.57, *p*-value < 0.001).

1. **Graphic integrity across countries**

We conducted a Pearson’s Chi-squared test to compare if bar charts with proportional ink violation are more likely from certain countries compared to bar charts without proportional ink violation. Our result show that some countries are more likely to have bar charts with proportional ink violation (Pearson’s Chi-squared test (based on 2000 replicates), X-squared (17) = 151.13, *p*-value < 0.001).

1. **Graphic integrity across year**

We conducted a Pearson’s product-moment correlation test to examine if bar charts with proportional ink violation are increasing or decreasing among the sample publications in the past. Our results show that there is no correlation between the likelihood of having proportional ink violation and year of publication (Pearson’s product-moment correlation test, r = -0.11, N= 9, *p*-value = 0.74).

1. **Regression**

We run a multivariate logistic regression analysis on annotation pipeline samples relating journal ranking, research field, citation count, author seniority (h-index), year of publication, number of affiliations, and country of affiliation. The model's explanatory power is weak (Tjur's R squared = 0.02). The results more worth mentioning are significantly higher relationship between some fields (psychology: standardized coefficient 1.03 (standard error=0.28), t(3582) = 5.19, p<0.001) and lower relationship between some countries (e.g., Japan: standardized coefficient -1.04 (standard error = 0.52), t(3582) = -2.01, p = 0.044) to proportional ink violations.
