# Supplementary material for: Graphical integrity issues in open access publications: Detection and patterns of proportional ink violations
Source: PLoS Comput Biol. 2021 Dec 13;17(12):e1009650. doi: 10.1371/journal.pcbi.1009650 (PMC8700024; doi:10.1371/journal.pcbi.1009650)
Supplement: Attachment — Submitted filename: PCOMPBIOL-D-21-01149-1(1).pdf [file pcbi.1009650.s009.pdf]

PLOS Computational Biology

Graphical integrity issues in open access publications: detection and patterns of proportional ink violations

--Manuscript Draft--

**Manuscript Number:**	PCOMPBIOL-D-21-01149
**Full Title:**	Graphical integrity issues in open access publications: detection and patterns of proportional ink violations
**Short Title:**	Graphical integrity issues in open access publications
**Article Type:**	Research Article
**Keywords:**	graphical integrity in science; large-scale study; science of science
**Abstract:**	Academic graphs are essential for communicating complex scientific ideas and results. To ensure that these graphs truthfully reflect underlying data and results, visualization researchers have proposed several principles to guide their creation process. However, it is unknown the extent of violations of these principles in academic publications. In this work, we develop a deep learning-based process to accurately measure violations of the proportional ink principle—one of the most basic and simplest data visualization rules—and apply it to analyze a large sample of 300K open access figures. Our results reveal that graphical integrity issues are significantly more prevalent in some types of journals, research fields, and regions of the globe. Additionally, we found no increase in integrity violations over time and seniority. Apart from openly releasing our large annotated dataset and technique, we discuss how these kinds of analyses should be part of the peer-review post-publication processes.
**Additional Information:**	
**Question**	**Response**
**Financial Disclosure**<br><br>Enter a financial disclosure statement that describes the sources of funding for the work included in this submission. Review the submission guidelines for detailed requirements. View published research articles from *PLOS Computational Biology* for specific examples.<br><br>This statement is required for submission and **will appear in the published article** if the submission is accepted. Please make sure it is accurate.	HZ, TYH, DEA were partially funded by the ORI HHS grants ORIIR180041 and ORIIIR190049.<br>The funders had no role in study design, data collection and analysis, decision to publish, or preparation of the manuscript.

Powered by Editorial Manager® and ProduXion Manager® from Aries Systems Corporation

**Unfunded studies**<br>Enter: *The author(s) received no specific funding for this work.*<br><br>**Funded studies**<br>Enter a statement with the following details:<br>• Initials of the authors who received each award<br>• Grant numbers awarded to each author<br>• The full name of each funder<br>• URL of each funder website<br>• Did the sponsors or funders play any role in the study design, data collection and analysis, decision to publish, or preparation of the manuscript?<br>• **NO** - Include this sentence at the end of your statement: *The funders had no role in study design, data collection and analysis, decision to publish, or preparation of the manuscript.*<br>• **YES** - Specify the role(s) played.<br><br>* typeset	
**Competing Interests**<br><br>Use the instructions below to enter a competing interest statement for this submission. On behalf of all authors, disclose any competing interests that could be perceived to bias this work—acknowledging all financial support and any other relevant financial or non-financial competing interests.<br><br>This statement **will appear in the published article** if the submission is accepted. Please make sure it is accurate. View published research articles from *PLOS Computational Biology* for specific examples.	The authors have declared that no competing interests exist.

Powered by Editorial Manager® and ProduXion Manager® from Aries Systems Corporation

**NO authors have competing interests**<br><br>Enter: *The authors have declared that no competing interests exist.*<br><br>**Authors with competing interests**<br><br>Enter competing interest details beginning with this statement:<br><br>*I have read the journal's policy and the authors of this manuscript have the following competing interests: [insert competing interests here]*<br><br>* typeset<br><br>This statement is required for submission and **will appear in the published article** if the submission is accepted. Please make sure it is accurate and that any funding sources listed in your Funding Information later in the submission form are also declared in your Financial Disclosure statement.	
**Data and Code Availability**<br><br>From the time of publication, Authors are required to make fully available and without restriction all data and computational code underlying their findings. Please see our PLOS Data Policy page for detailed policy information, and our Code Sharing page for specific information on code sharing.<br><br>A **Data Availability Statement**, detailing where the data (and code, if applicable) can be accessed, is required at first submission. Insert your Data Availability Statement in the box below. The statement you provide **will be published in the article**, if accepted.<br><br>Please see the Data Reporting section of our submission guidelines for instructions on what you need to include in your Data Availability Statement.	All relevant data and code will be made available in a Github repository and Zenodo dataset.

If the data and code are all contained in your submission files, please state: *All relevant data are within the manuscript and its Supporting Information files.*<br><br>**PLOS allows rare exemptions to address legal and ethical concerns.** If you have legal or ethical restrictions, please detail these in your Data Availability Statement below for the Journal team to consider.	

Powered by Editorial Manager® and ProduXion Manager® from Aries Systems Corporation

Manuscript

Click here to access/download;Manuscript;Graphical Integrity In Open Access Journals and Its Patterns.pdf

Graphical integrity issues in open access publications: detection and patterns of proportional ink violations

Han Zhuang, Tzu-Yang Huang, Daniel E. Acuna
School of Information Studies
Syracuse University

Abstract

Academic graphs are essential for communicating complex scientific ideas and results. To ensure that these graphs truthfully reflect underlying data and results, visualization researchers have proposed several principles to guide their creation process. However, it is unknown the extent of violations of these principles in academic publications. In this work, we develop a deep learning-based process to accurately measure violations of the proportional ink principle—one of the most basic and simplest data visualization rules—and apply it to analyze a large sample of 300K open access figures. Our results reveal that graphical integrity issues are significantly more prevalent in some types of journals, research fields, and regions of the globe. Additionally, we found no increase in integrity violations over time and seniority. Apart from openly releasing our large annotated dataset and technique, we discuss how these kinds of analyses should be part of the peer-review post-publication processes.

1. Introduction

Scientific communication needs the accurate transmission of results. This goal is best achieved when research findings are conveyed clearly and honestly without integrity issues. Intentionally or unintentionally, however, scientific publications can contain fabrication, falsifications, or plagiarism (Office of Research Integrity, 2021). Research integrity issues waste time and valuable resources for scientists and the public at large (Sox & Rennie, 2006). While these behaviors may seem rare and extreme, they are more prevalent than we might expect. For example, a survey of researchers revealed that around 9% of them have observed or have evidence of others' research misconduct (Titus et al., 2008). We as scientists should guard against these issues by attempting to actively detect and correct them.

Research integrity encompasses a broad range of media such as text, data, and graphs. Text and data are usually accessible in a machine-readable format and therefore are relatively simpler and better studied compared to graphs and images. Still, studies driven by human examination have found that common manipulations of graphs and images can produce serious integrity violations (Beck, 2016). Some of these manipulations, such as adjusting the size and brightness of images, are very common and might be misused. Several guidelines have been proposed to avoid these problems (Cromey, 2010; Jordan, 2014).

While there are also guidelines for integrity in scientific graphs (Tufte, 2001), the prevalence of graphical integrity issues in this domain is relatively unknown. Scientific graph problems can have negative

consequences especially considering how much the other scientists and the public trust scientists. For example, truncating the y-axis of bar charts is a simple yet way of exaggerating differences among groups but it is hard to detect and has detrimental effects. Graphical integrity issues such as this can successfully deceive the receivers of the information (Yang et al., 2021), affecting their ability to make decisions (Larkin, 1990). Even if the readers are trained to detect manipulations, they can still be misled (Raschke & Steinbart, 2008). To the best of our knowledge, we do not know the seriousness of graphical integrity issues among scholarly publications. With the availability of new datasets about scientific figures and open access publications, we have unprecedented opportunities to understand the extent of these issues.

Researchers have proposed automated techniques that scale to detect violations of research integrity. For example, researchers have developed computer techniques to detect image tampering and image reuse at scale (Acuna et al., 2018; Xiang & Acuna, 2020). These studies reveal the prevalence of image fabrications in science beyond small samples of images (Bik et al., 2016a). Graphical integrity in graphs is as important as these other kinds of integrity issues. However, we lack techniques and curated datasets to create methods to detect them appropriately.

This study develops a novel method based on deep learning to detect graphical integrity issues automatically. We specifically focus on proportional ink principle violations. Our framework is general enough that it can be adapted to other kinds of graphical integrity issues (i.e., data-ink principle). Armed with this highly accurate technique (AUC > 0.9), we seek to answer how common these kinds of problems are in academia and whether there are systematic differences among researchers, journals, fields, and countries. Further, we examine whether graphical integrity issues "run in the family": does being a co-author of an article with issues in the past predict problems in the future? Finally, we discuss applications of these ideas beyond the proportional ink principle and graphical integrity.

2. Literature review

2.1 Graphical integrity

In visualization design, graphical integrity requires designers to create graphs reflecting the actual data. There are several graphical integrity principles studied in the literature, and two of the most common are the principle of proportional ink and the principle of data-ink (Tufte, 2001). The principle of proportional ink states that "the representation of numbers, as physically measured on the surface of the graphic itself, should be directly proportional to the numerical quantities represented" (Tufte, 2001). The principle of data-ink states that the graphs should use most of the ink to present the data instead of using it to show the background and aesthetic features. According to the literature on visualization, violations of these principles can lead viewers to misunderstand the underlying data and results (Yang et al., 2021).

A field that has extensively studied graphical integrity is business. Researchers in this area have investigated problems in annual reports and other forms of financial information, uncovering graph distortions and truncations of bar charts and other types of graphs (Beattie & Jones, 1992; Courtis, 1997). Some researchers have examined the effects of these distortions and found they can greatly mislead viewers (Beattie & Jones, 2002). We would expect that these misleading potentials would translate into other fields beyond business.

Studies about graphical integrity issues in science are, in contrast, much less common. These issues, however, can be part of more significant problems such as misinformation in science (West & Bergstrom, 2021), and therefore are essential to understand. For example, researchers have found inadequate reporting of research in the news (Gonon et al., 2011), exaggerations of research from press releases (Sumner et al., 2014), and predatory publishing of research in some open access journals (Grudniewicz et al., 2019; Shen & Björk, 2015). Although we can find some graphical integrity issues in news and scientific publications (C. T. Bergstrom & West, 2020b), large-scale studies about graphical integrity in science are lacking.

2.2 Chart mining

Researchers can investigate the graphical integrity of science through automated computational techniques. Chart mining is a research field dedicated to developing strategies to extract figures from documents, recognize subfigures from compound figures, classify charts into categories, and extract data from them. We review some standard techniques in chart mining relevant to our research.

Chart extraction. Charts can be embedded in documents and not available separately. Chart extraction is the process of extracting them from these documents. Some of these chart extraction techniques exploit metadata available in PDFs or are based on computer algorithms to guess the location and size of graphs within a document (Clark & Divvala, 2016; Li et al., 2019). With the development of neural networks, the accuracy of these algorithms has increased substantially (Siegel et al., 2018). Chart extraction is a relatively mature field.

Subfigure separation. Even after we have extracted figures from publications, some of them need preprocessing before analysis. Subfigure separation is one typical preprocessing step that removes the panels present in an image (García Seco de Herrera et al., 2016). Of course, not all analyses need panel separation because compound figures can focus on some research in themselves. Researchers have proposed methods to separate subfigures from compound ones using traditional computer vision algorithms such as edge-based detection of spaces between panels (Taschwer & Marques, 2018, 2016). Similar to what has happened with chart extraction, subfigure separation has benefited from new deep learning techniques trained on large annotated compound figures (S. L. Lee & Zare, 2018). While there are still challenges for this step, subfigure separation is mature enough that can be applicable at scale.

Chart classification. Different types of charts serve different kinds of analyses (Shneiderman, 2003; Tufte, 2001). For example, a pie chart might represent proportions among components, and a line chart might represent temporal dynamics of such components. Chart classification is usually part of an analysis pipeline, and therefore classification performance has a cascading effect. Researchers have used a range of techniques from traditional computer vision algorithms (Prasad et al., 2007) to deep learning techniques (Chagas et al., 2018) to perform this task. This chart mining step is relatively less developed than the previous ones.

Data extraction. A bar chart, plot, and pie chart panels might contain valuable information to extract—charts are images without data. Data extraction is the most complex step as it attempts to reverse engineer the data used to produce such panels. It usually combines text detection, optical character recognition, keypoint analysis, and other advanced computer vision methods (Al-Zaidy & Giles, 2017) and neural networks (Choudhury et al., 2016; Vassilieva & Fomina, 2013). Depending on the type of graph, specialized

data extraction techniques (e.g., bar charts or pie charts use different methods; see (Nair et al., 2015)). Data extraction is thus an active area of research.

2.3 Chat mining for graphical integrity

One major step for evaluating graphic integrity automatically is to understand graphs with computer techniques. Several studies have attempted to achieve this step. For example, one study showed that we could tell the field of manuscript just by looking at its figures (P.-S. Lee et al., 2018). Another study developed machine learning techniques to extract information from graphs, including bar charts, line plots, and scatter plots (Cliche et al., 2017; Poco & Heer, 2017). In addition, powered by deep learning, some researchers have shown how these techniques can accurately extract the raw data used to construct a graph (Luo et al., 2021). There are, however, significant challenges left when deadline with low resolution images. Similarly, each domain and scientist can produce completely different charts to represent the same information, making chart mining for graphical integrity challenging.

With the convergence of new chart mining techniques, more extensive datasets, and more computational power, the automation of graphical integrity investigation is bound to explode in possibilities. Yet, to the best of our knowledge, there is no study about the automated detection of graphical integrity issues in science.

2.2 Datasets about science

In the recent past, we lacked the datasets necessary to analyze science. For example, citations were only available from the Web of Knowledge from Web of Science (now Clarivate Analytics) dating back to 1995 only. However, we now have access to a much broader set of information pieces about science. These datasets include citations and other artifacts (e.g. (Lo et al., 2020; Sinha et al., 2015)). For example, and due to the push for open access publications, we have unprecedented access to full text, citation contexts, figures, tables, and datasets (*PMC Overview*, 2021). These new datasets open up a wealth of opportunities to understand graphical integrity issues.

3. Results

3.1 Evaluation of our automated graphical integrity detector

3.1.1 Labeled dataset for evaluation

We labeled images from the large Pubmed Central (PMC) Open Access Subset collection (see Materials and Methods). The PMC Open Access Subset is a part of the total collection of articles in PubMed Central (denoted as PMC). We perform the traditional steps of chart mining from an initial 50,000 random samples of figures (see Literature Review and Fig. 1). The classification method detected 8,001 panels or figures as bar charts. Two annotators (co-authors) manually annotated these bar charts as having proportional ink principle violations or not. We found 356 graphs with integrity issues and 7,645 graphs without them. Because our prediction method relies on automatically extracting features from the bar charts (e.g., tick labels, the start of the y-axis, etc.), this extract was not always successful. In particular, among the 8,001

annotated bar charts, our method could only extract features from 4,834 of them (with integrity issue: 265, without integrity issue: 4569).

3.1.2 Evaluation of the method for estimating graphical integrity issues

We used Area under the ROC curve (denoted as AUC) with stratified 5-fold cross-validation to evaluate the performance of our graphical integrity evaluator. We additionally investigated the precision and recall of our method. Precision is computed as Eq. (1), which is the ratio of positive data points with true positive predictions to all positive data points (Manning et al., 2008). In our evaluation, true means the image does have an integrity issue, and true positive means the image has an graphical integrity issue and our method also predicts the image has this issue; false positive means the image does not have an integrity issue, but our method predicts the image has an issue. And false negative means the image does have graphical integrity issues, but our method predicts it has no graphical integrity issues. The average precision across folds is 0.77 with 0.0209 standard error. The recall is the ratio of true positive data points to all true data points (Eq. (2)). False Negative means the image has an integrity issue, but the predicted class is not having problems. The average recall across folds is 0.37 with 0.0207 standard error. Additionally, the AUC indicates the ability of our detector to classify two classes, and the average AUC across folds is 0.917 with a standard deviation of 0.02 (Manning et al., 2008).

$$Precision = \frac{True\ Positive}{True\ Positive + False\ Positive} \tag{1}$$

$$Recall = \frac{True\ Positive}{True\ Positive + False\ Negative} \tag{2}$$

3.2 Graphical integrity in open access journals

With this automated graphical integrity evaluator, we can examine graphical integrity issues at scale. In the present study, we apply this evaluator to open access journals to investigate the commonness and patterns of graphical integrity issues in science. In the following subsections, we first present a descriptive analysis of our data. Then, we offer analysis by countries, research fields, years, seniority of researchers, and the journal's impact. We combined the 50,000 figures from the labeled dataset with another 250,000 computer-annotated figures to analyze.

3.2.1 Representation quality of unlabeled data vs. labeled data

We first ensure that the unlabeled data that we analyze is similar to the manually labeled dataset. We compare both datasets across various features related to publication, authors, journals, country, fields, and year. Because we randomly sampled both datasets, we expected them to be similar. Indeed, we found that the unlabeled and labeled distributions are nearly identical (Fig. 2).

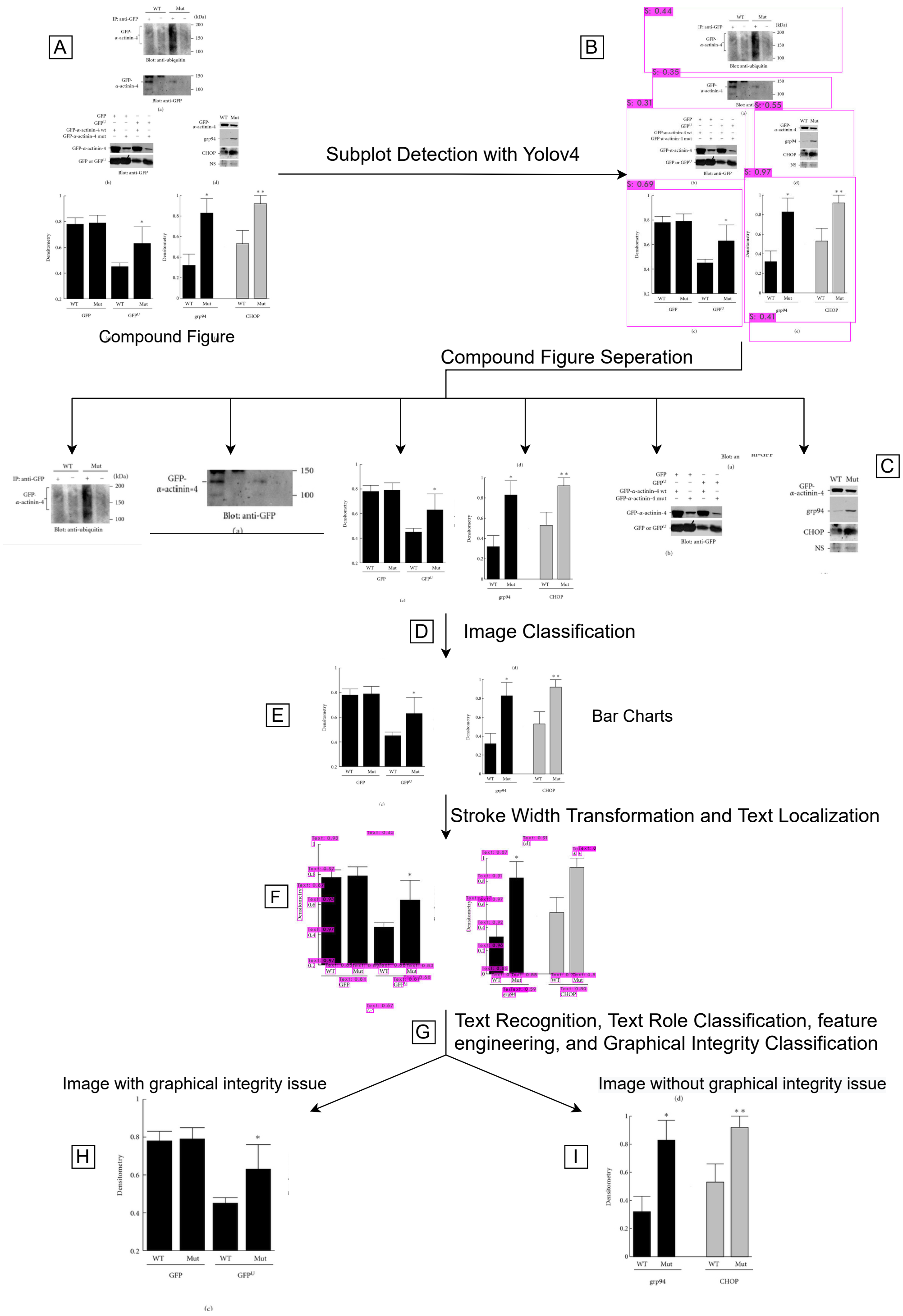

Figure 1. An example process for predicting violations of the proportional ink principle. A. Input image representing a scientific figure. Pubmed Open Access subset provides figures already extracted from the publications. B. Subplot extraction using the YOLO deep learning architecture (Bochkovskiy et al., 2020) trained on the hand-annotated dataset (see Materials and Methods). C. Each subplot is extracted from the input image. D. Subfigure plot classification where only bar charts are extracted (E). For each bar chart, we detect a set of low-level features (F), which are later used for predicting whether a bar chart is violating the proportional ink principle (H, yes) or not (I, not).

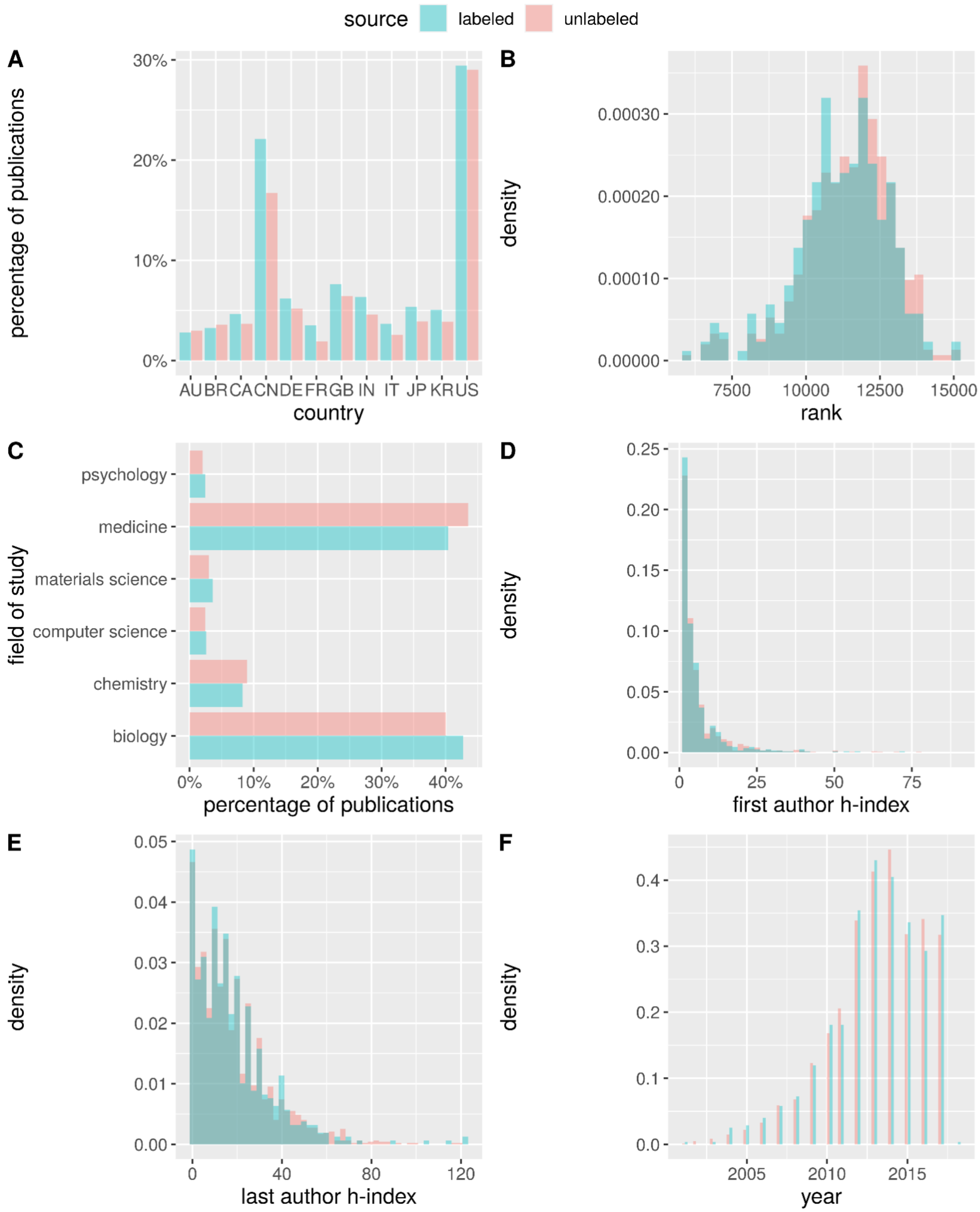

Figure 2. Labeled vs. unlabeled data. Both datasets are nearly identical therefore validating our prediction step. (A) compares the percentage of publications in each country of affiliations (having more than 20 publications). (B) is the histogram of the rank of the journal of publications. (C) compares the percentage of publications in each field of journals (having more than ten publications) (D) is the histogram of the h-index of the first author of publications (E) is the histogram of the h-index of the last author of publications. (F) is the histogram of the year of publications.

3.2.2 The commonness of graphical integrity issues in open access journals

Among our sample of images, we found 265 graphical integrity issues in 4,834 human-annotated bar charts that can be processed by our algorithm (6.5% per publication with bar chart, 5.5 % per bar chart) (Not all bar charts can have their features extracted for our algorithm. See Methods and Materials.) We additionally predict that there are 479 graphic integrity issues of 20,666 bar charts (3.6 % per publication with bar chart, 2.3 % per bar chart). A summary display of the data is in Table 1 in Materials and Methods. We found that the percentage of graphical integrity issues is significantly larger in human-annotated than automated prediction bar charts (the p-value is less than 0.05 for both the ratio per publication with a bar chart and the ratio per chart by Fisher's exact test). This difference suggests that our method is significantly under-estimating the extend of graphical integrity issues.

3.2.2 Graphical integrity differences by seniority

We now investigate if there is a relationship between seniority and the ratio of graphical integrity issues. To examine this research question, we conducted a correlation test between the likelihood of having graphical integrity issues and the h-index of the first author of publications and the last author of publications. (We acknowledge that the h-index of researchers might not fully reflect the seniority of researchers.) We found no statistically significant correlation for first author (r = 0.024, N = 2,530, p-value = 0.22) or last author (r = 0.018, N = 3,179, p-value = 0.31). Similarly, we compared the h-index of the first authors with graphical integrity issues to those without and found no significant difference (Welch's t-test, $t(80.55) = 0.52$, p-value = 0.61). We did a similar analysis comparing the last authors and found no statistical significance (Welch's t-test, $t(94.25) = 0.98$, p-value = 0.33).

3.2.3 Graphical integrity across the impact of journals

High-impact journals are generally expected to publish rigorous research articles. Graphs as a part of research articles should also be accurate, but we do not know if the review of high-impact journals weeds out low-quality graphs. We tested the correlation between the rank of journals and journals' likelihood of having at least one article with graphical integrity issues. To measure this likelihood, we compute the maximum likelihood of a graph integrity per journal. A Pearson correlational analysis revealed that the higher the ranking (here, "higher" means closer to 1), the more likely to have articles with graphical integrity issues (r = -0.16, N = 530, p-value < 0.001). Similarly, we compared the difference between the rank of journals of articles with graphical integrity issues and the rank of journals of articles without graphical integrity issues (Welch's t-test, $t(88.97) = -3.6099$, p-value < 0.001).

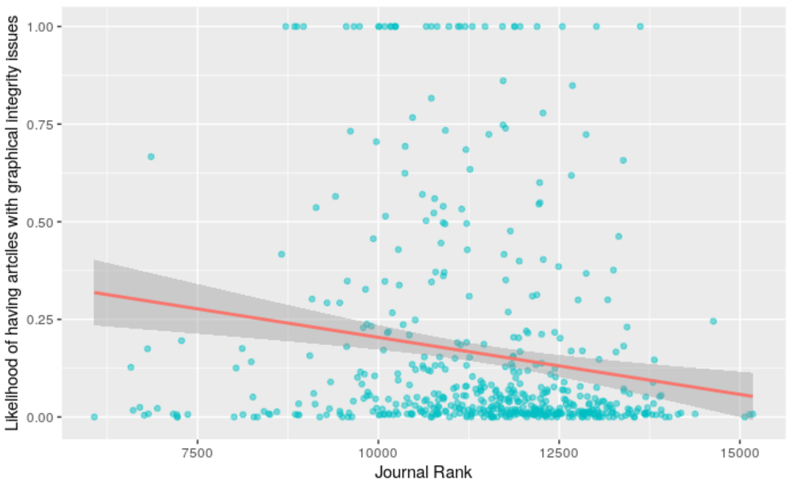

Figure 3. Correlation between journal rank and the likelihood of having articles with graphical integrity issues. The orange line represents the estimation of linear regression.

3.2.4 Graphical integrity across research fields

Graphical representations of information vary substantially across fields. Thus, proportional ink principles might be followed at different rates by different fields. To understand these differences, we compared the likelihood of having graphical integrity issues across research fields. As we can see in Figure 4, different research fields have different commonness of graphical integrity issues ($F(6, 3084) = 3.68$, *p*-value = 0.001). A posthoc analysis (Tukey's HSD) reveals that computer science contains violations of proportional ink principles significantly more frequently than material science (*p*-value = 0.01), chemistry (*p*-value = 0.004), and biology (*p*-value = 0.002).

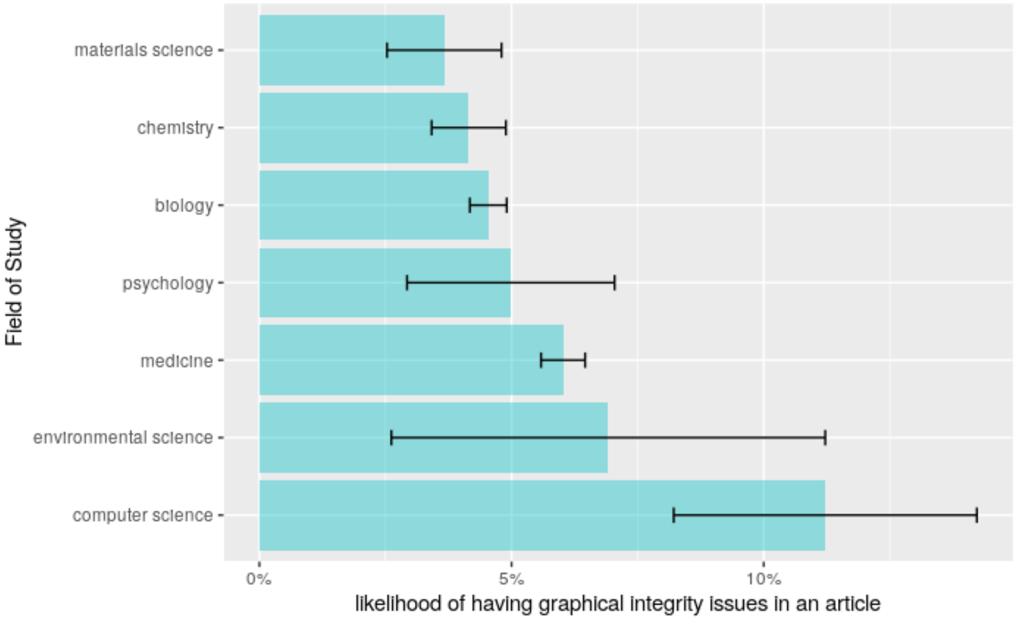

Figure 4. The likelihood of having graphical integrity issues across each research field

3.2.5 Graphic integrity across countries

As previously shown, there are significantly different attitudes toward research integrity across countries (Bik et al., 2016b). Here, we examined the likelihood of having articles with graphical integrity issues for authors from different countries and found that there is a statistical difference between authors from countries ($F(12, 12591) = 10.34$, p-value < 0.001). A posthoc analysis (Tukey's HSD) reveals that France is more likely to have graphical integrity issues than Japan (p-value = 0.019), China (p-value = 0.003), and Korea (p-value < 0.001). Also, Italy is more likely to have graphical integrity issues than Japan (p-value < 0.001), China (p-value < 0.001), and Korea (p-value < 0.001). The United States is more likely to have graphical integrity issues than Japan (p-value = 0.002), China (p-value < 0.001), and Korea (p-value < 0.001). We further aggregate the likelihood of having graphical integrity issues per article for each country in Fig. 5.

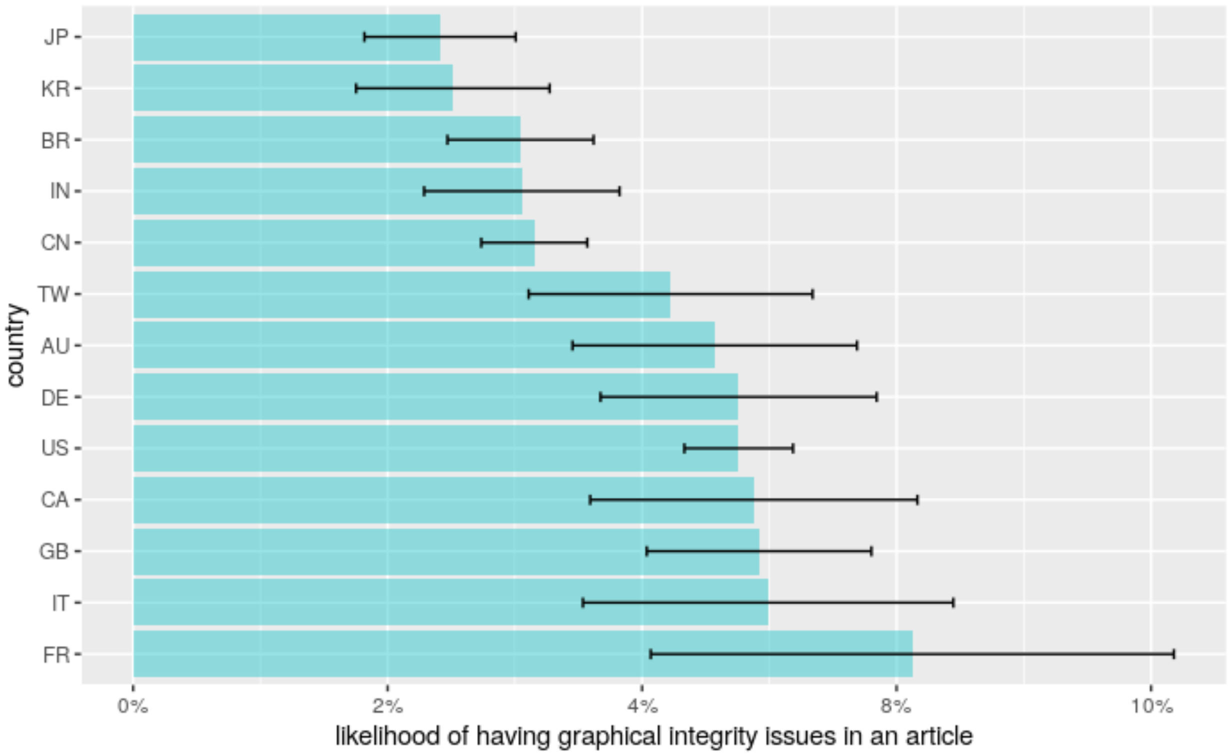

Figure 5. The likelihood of having graphical integrity issues across each country

3.2.6 Graphic integrity across years

Whether publishers and research communities have been aware of graphical integrity issues in the past and taken actions to protect graphical integrity is another unknown research question. We computed the correlation between the likelihood of having graphical integrity issues and publication year to examine if graphical integrity issues are decreasing or increasing over time (see Fig. 6). We did not find a relationship between these quantities suggesting that graphical integrity issues have been consistent ($r = 0.0005$, N = 18, p-value = 0.99).

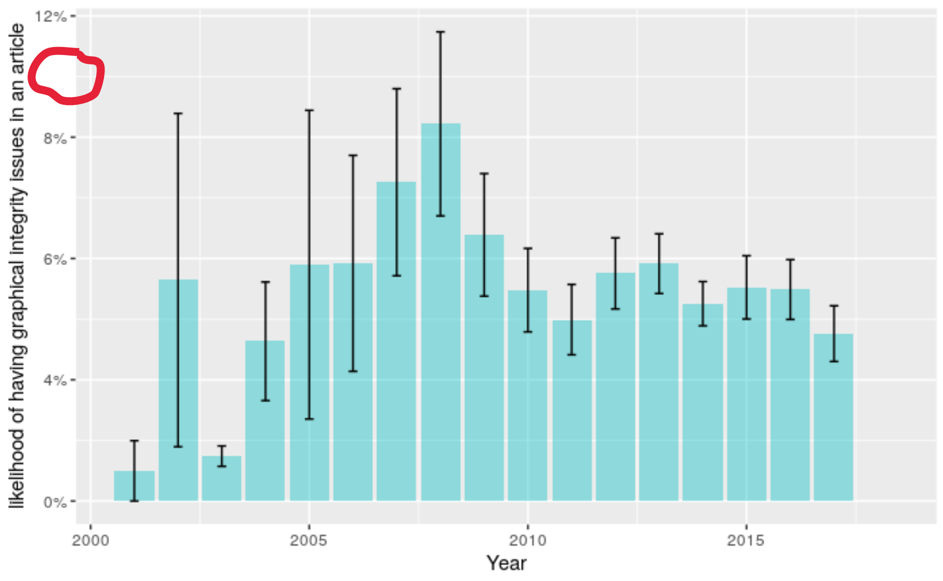

Figure 6. The likelihood of having graphical integrity issues across each year

3.2.7 Within-author integrity trends

We now examined whether having experienced graphical integrity issues in the past is predictive of graphical integrity issues in the future. Of the graphs produced by authors who had graphical integrity issues in the past, we analyzed 301 bar charts in the future and found that only 5 of them had problems. Of the graphs produced by authors who did not likely have graphical integrity issues in the past, we analyzed 568 bar chart features and found that 6 of them had problems. According to a Fisher's Exact Test, the difference between these two groups is not statistically significant (p-value = 0.53). We then analyzed at the author level. We found that 7 out of 218 authors with issues in the past had problems in the future; we found that 10 of 423 authors with potential issues had problems in the future. Again, Fisher's Exact Test's difference between these groups of authors is not statistically significant (p-value = 0.61). These results suggest that graphical integrity issues do not seem to "run in the family": having issues in the past might not make an author have problems in the future.

4. Materials & Methods

4.1 Materials

4.1.1 Pubmed Open Access and annotations

Pubmed Open Access is a subset of Pubmed Central, consisting of thousands of journals in biomedical research (*PMC Overview*, 2021). To train our machine learning-based graphical integrity evaluator, we annotated one subset of collected images mentioned in the previous subsection (after compound figure detection and subfigure separation) as graphical integrity issues and others (see Table 1). We consider images as graphical integrity issues if they violate the principle of proportional ink. The specific rules are: a bar chart's y-axis should start from zero, have one scale, and not be partially hidden (C. T. Bergstrom &

West, 2020a; Tufte, 2001). We annotate images as *others* if they follow all three rules, do not have a full y-axis or x-axis, or are not bar charts.

All PMC papers	7 million articles	
	Human annotations	Predictions
Images	50,000	250,000
Papers*	29,901	68,265
Bar charts	8,001	35,481
Processable Bar charts	4,834	20,666
With / Without graphical integrity issues	265 / 4,569	479 / 20,187

**Image (and not the paper) was the unit of sample and analysis*

Table 1. Summary of human-annotated sample and prediction sample. Both sets are similar, as demonstrated by analysis in Fig. 2.

4.1.3 Datasets for image processing techniques

To automate graphical integrity evaluation, we need image processing techniques to obtain information from images. This study has six image processing steps: compound figure classification, subfigure separation, image classification, text localization, text recognition, and text role prediction (see the summary of training data and validation data in 2).

Data for testing within-author integrity issues. After labeling images, we analyzed the publications of these authors in the following three years after the first year we annotated. Based on the labeled data, we found 218 authors who produced graphs with integrity issues in their publications and analyzed 301 bar charts in their following publications. Also, there are 4780 authors which we did not find graphical integrity issues in our labeled data. Due to the unbalanced amount between the two groups, we randomly selected 3000 images from the following publications and analyzed 568 bar charts.

We now describe the features used in our statistical analyses:

Seniority of researchers. As the research experience of researchers grows, some researchers might be aware of graphical integrity and want to avoid graphical integrity issues. Thus, graphic integrity issues might be less common among senior researchers, for they have more education and knowledge. If this hypothesis holds, then it might imply that researchers can learn graphical integrity over time by themselves. If not, research communities might need more education about this topic to reduce such problematic practices. To examine this hypothesis, we collected researchers' h-index to proxy their seniority for the analysis.

Impact of journals. High-impact journals usually have a strict standard for their publications. It is natural to expect high-impact journals to publish more rigorous research findings. However, whether high-impact journals value graphic integrity is unknown. Thorough research should make sure its visualizations convey

accurate information to readers. If not, the journal publishers might need to include graphical integrity into their review standard. To examine this research question, we collected the rank of journals of articles in our sample.

Research fields. Different disciplines have their traditions, such as citation format and writing style. Similarly, other disciplines might have additional requirements for graphical integrity. However, this is a new and important research question because the publishers might want to enhance the review of graphical integrity for specific research fields. To address this question, we collected the field of study of journals of articles in our sample.

Countries. Education and culture vary from country to country. But we do not know if some countries have stricter graphic integrity. This research question can help researchers to know which country needs more protection of graphical integrity. Thus, we collected the country of the author affiliation of articles in our sample.

Year of publication. Science is changing with our research policy, education, and society over time. The time-series analysis of science helps researchers to understand the direction of new science. Similarly, graphical integrity over time can help the public to know if the awareness of graphical integrity changed in the past. To answer this research question, we collected the year of publications of articles in our sample.

4.2 Methods

4.2.1 Image preprocessing

The goal of image processing is to obtain information from images for feature engineering and classification of graphical integrity issues. Our image preprocessing includes the following steps:

Compound figure classification. Compound figures are standard in academic papers (e.g. (P. Lee et al., 2018)), but usually, graphical integrity issues are defined on non-compound figures. Therefore, we fine-tuned a convolutional neural network (Resnet-101v2, pre-trained on ImageNet) to classify figures into compound figures and non-compound figures.

Compound figure separation. After we have compound figures, we need to separate compound figures into subfigures because subfigures in compound figures might also have graphical integrity issues. To achieve subfigure separation, we trained a convolutional neural network (YOLO v4, pre-trained on MS COCO dataset) to localize subfigures in compound figures (Bochkovskiy et al., 2020).

Image classification. This study focuses on bar charts because the violation of the principle of proportional ink is a widely-accepted graphical integrity issue for bar charts. Thus, we generated charts (bar charts, line charts, scatter charts, heatmap charts, box charts, area charts, radar plots, maps, pie charts, tables, Pareto charts, Venn diagrams, and violin charts) and collected diagnostic figures from ImageCLEF 2016 competition (García Seco de Herrera et al., 2016). Then we fine-tuned a convolutional neural network (Resnet-101v2, pre-trained on ImageNet) to classify figures into categories.

Text localization. To predict if a bar chart has graphical integrity issues, we also need to know the texts on images for our classification. Therefore, we fine-tuned a convolutional neural network (YOLO v4, pre-

trained on MS COCO dataset) to detect or localize texts on academic figures, preprocessed with Stroke Width Transformation (Bochkovskiy et al., 2020; Epshtein et al., 2010).

Text recognition. More than the location of texts on graphs, we also need their content. Thus, we used Tesseract to recognize the content of texts based on the predicted locations of texts from text localization. We used one fine-tuned Tesseract model of English texts for this task.

Text role classification. Also, we need to know the role of texts on each figure for our analysis and thus use one open-source model to predict the role of texts on academic figures, based on the geometric information of texts on graphs (Poco & Heer, 2017).

Image preprocessing validation. To evaluate the performance of previous tasks, we used public datasets and our own generated datasets (when we could not find public datasets). The summary of our performance and benchmarks is summarized in Table 2.

**Steps**	**Training data**	**Validation Data**	**Our Performance**	**Benchmark**
Compound Figure Detection	ImageClef Medical 2016 Compound Figure Task	ImageClef Medical 2016 Compound Figure Task	Accuracy: 92%	Accuracy: 92% (Top one team in ImageClef Medical 2016)
Subfigure Separation	ImageClef Medical 2016 Compound Figure Task	ImageClef Medical 2016 Subfigure Separation Task	Score: 83%	Score: 84% (Top one team of ImageClef Medical 2016)
Chart Classification	Our generated charts	Revision data (not fully available)	Accuracy: 100%	Accuracy: 80%[1]
Text Localization	Localization from Arxiv papers through pdffigures	Our generated charts	F1: 76%	F1: 88%[2]
Text Recognition	No training data (Used an open-source fine-tuned model)	Our generated charts	F1: Exact 82% / Edit 90%	F1: Exact 95%/ Edit 98%[2]
Text Role Classification	No training data (Used an open-source fine-tuned model)	Our generated charts	F1: 80%	F1: 100%[2]

Table 2. Summary of Training and Validation for Image Preprocessing. [1](Savva et al., 2011), [2](Poco & Heer, 2017)

4.2.2 Feature engineering

One way to build an automatic graphic integrity issues classifier is to apply classifiers from machine learning. A machine learning-based classifier needs features from data instead of the raw data. Thus, we conducted a feature engineering step from our image preprocessing (Table 3). Because the principle of proportional ink requires the ratios between the numeric value and the area of each bar to be the same in the same bar chart, the violation of this principle has the following symptoms for academic figures: bar charts with a non-zero y-axis and bar charts with a y-axis of multiple scales (also considering partially hidden y-axis). These two kinds of bar charts all violate the principle of proportional ink. Although bar charts with a log scale also violate the principle of proportional ink, we do not consider these bar charts as graphical integrity issues because log-scale is widely accepted in academic figures (C. Bergstrom & West, 2021). To examine if one bar chart has the above symptoms of graphical integrity issues, we transform the information from our image preprocessing into features for the final classification. The first two features are derived directly from the principle of proportional ink for bar charts. However, based on our experiments, these two features are not enough to detect graphical integrity issues because our image preprocessing might introduce errors to these two features. For example, the y-axis label might not be recognized by our text localization model. Thus, we created other features to consider the errors from our image preprocessing.

No	Feature Description	Reason
1	The value of the lowest y-axis label on the y-axis (detected or inference from y-axis)	The lowest y-axis label should be zero
2	The increasing rate between each pair of y-axis labels	The scales of y-axis should be consistent across each pair of neighbor y-axis labels
3	If we need to inference the lowest text on the y-axis	If the lowest label on the y-axis is far from the x-axis, then we might ignore the actual lowest label on the y-axis
4	If the y-axis has a mix of integer and float number	Tesseract might not perform well with float number, and thus the increasing rate in the y-axis might not be accurate
5	The probability of being texts	We prefer texts with a higher probability of being texts
6	The OCR confidences of texts on the y-axis	We prefer predictions of the content of texts with a higher confidence
7	The probability of being bar charts	Our classifier only classifies bar charts. Thus we prefer figures with a high probability of being bar charts

Table 3. Summary of features for proportional ink violation detection

4.2.3 Proportional ink principle evaluator

For an automatic graphical issue detector, rule-based methods are feasible when the features are highly correct. However, given the errors introduced from image processing, the texts and their roles on figures might not be correct. Thus, we took a probabilistic approach: the random forest model from machine learning. Our random forest model can predict if a figure is misleading or not based on the features of figures from our image processing techniques (see Fig. 7).

5. Discussion

In this article, we attempt to understand the extent of graphical integrity issues in scientific publications. We focused on violations of the proportional ink principle, one of the most basic visualization rules. We developed a method based on deep learning to detect these issues by learning from a large sample of human-annotated charts. Our approach achieved high accuracy, allowing us to apply it to an extensive set of open access publications. Our results suggest varying degrees of prevalence across fields, countries, journal rank. Overall, our manuscript provides one of the first looks into using automated methods to explore issues of graphical integrity at scale.

This study presents an automated method to detect the violation of the principle of proportional ink and shows the potential to automatically detect other graphical integrity issues. Researchers have relied on human experts in previous studies (Beattie & Jones, 2002; Courtis, 1997). Human experts can detect graphical integrity issues with very high accuracy, but the speed of annotation can be slow. Our automated method can examine various kinds of images at scale and detect graphical integrity issues with a relatively high accuracy to remedy this shortcoming. Moreover, the components in this method can address subfigure separation and compound figure classification for other graphical integrity issue investigations. Our process can complement other work in the field while being relevant and similarly effective (S. L. Lee & Zare, 2018; Taschwer & Marques, 2018, 2016). Beyond graphical integrity issues, our results could be part of other kinds of analysis. For example, studies of the quality of visual communications using human perception and learning (Shukla & Samal, 2008). Thus, our method opens the door to new kinds of automated analysis of publications for pre-print services, peer review, and post review.

Some researchers have found that research integrity issues have various frequencies across countries and journals (Bik et al., 2016b). We have discovered that graphical integrity issues vary across journal rank, research fields, and affiliation countries. Our result implies that graphical integrity issues might be more common in specific groups of researchers and various research communities. According to our results, articles published in a higher-ranked journal are more likely to have graphical integrity issues. This research finding might be generalizable to a broader range of journals because high-impact journals favor significant research findings (Murtaugh, 2002), and manipulations of graphs can exaggerate research findings for significance. According to this study, certain groups of articles are more likely to have such issues. Therefore, we can prioritize education, peer reviews, and post-publication corrections for these groups.

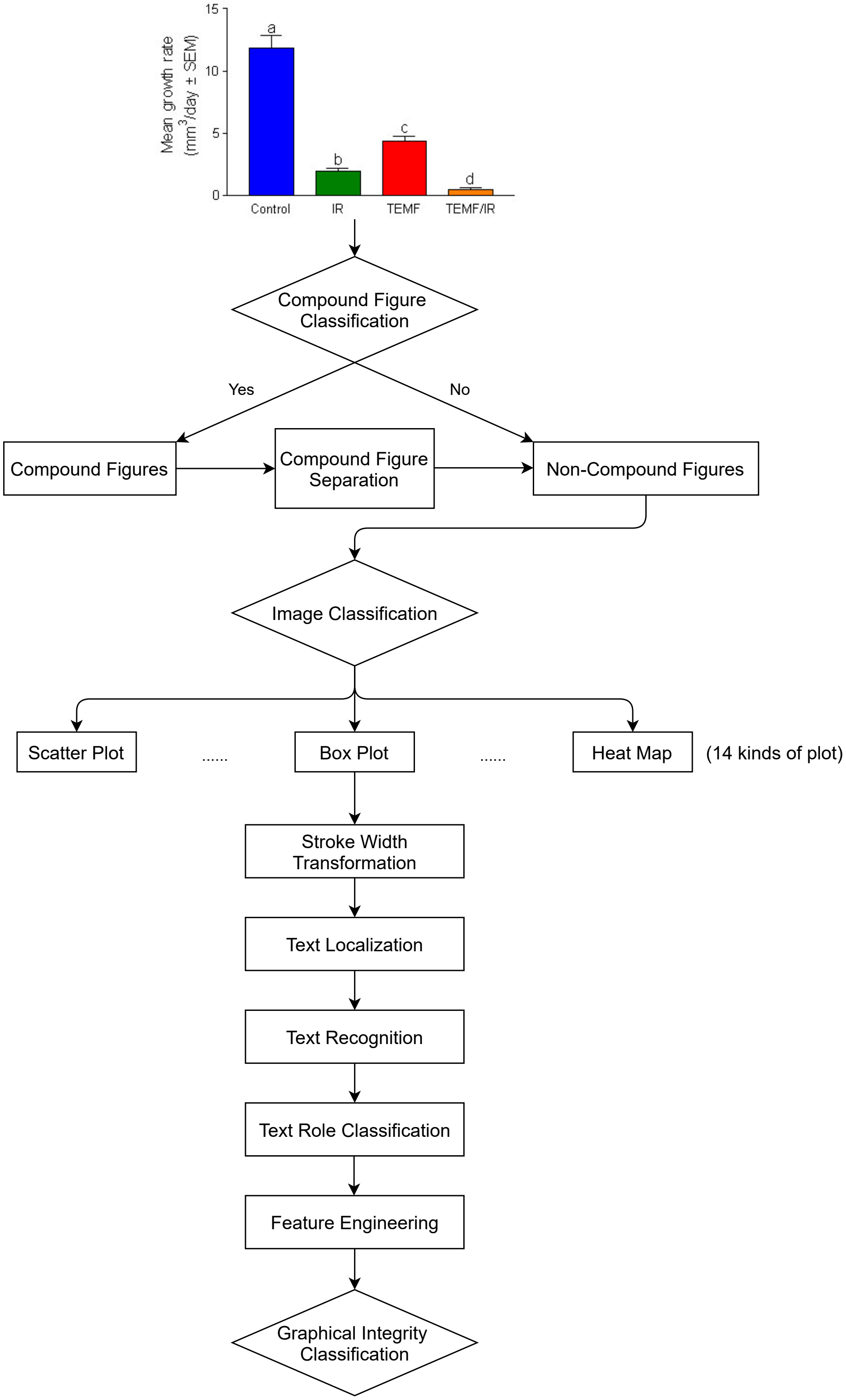

Figure 7. Flowchart of our graphical integrity classification. Refer to Fig. 1 for comparison.

There are several limitations of this study. First, this proposed method makes mistakes in compound figure classification, subfigure separation, and bar chart classification (see Table 2). Thus, some bar charts might

not be separated from compound figures correctly or might not be classified into bar charts correctly. In such cases, some bar charts are missed from the final classification of graphical integrity issues, and some graphical integrity issues are overlooked by this method. Second, this method might not localize all texts and recognize the contents of all texts correctly (see Table 2). In this scenario, our method does not have enough information about graphs to predict if one graph has graphical integrity issues (we could not process and predict all bar charts in our sample, see Table 1). We also think that there are limitations in our findings that we can improve in the future. First, our method can not detect all graphical integrity issues, and therefore we are underestimating their prevalence. In the future, we hope to incorporate this analysis with other kinds of integrity checks such as (Acuna et al., 2018; Xiang & Acuna, 2020). Second, in analyzing the association between the graphical integrity of articles and other features about articles, our sample does not cover some groups of articles in all journal rankings. This limitation might affect the trend that articles with graphical integrity issues are more likely to have a higher rank of journals. In the future, we will do a more hypothesis-driven sample where we will prioritize having a representative set of journal rankings, seniority, and other variables of interest. Improvements in these image analysis steps are an active area of research, and therefore should be improved in the future.

Publishers, funders, and scientists might consider incorporating graphical integrity into their peer review procedures and conduct post-publication corrections. Research communities could enhance their education about graphical integrity to all researchers to reduce the burden of publishers and reviewers on graphical integrity. These ideas are already part of research integrity initiatives around the world including COPE practices and the Hong Kong Principles (Committee on Publication Ethics, 2021; Moher et al., 2020).

6. Conclusion

This study presents a method to detect graphical integrity issues and research findings of graphical integrity issues in science. This method enables researchers to detect proportional ink violation at scale and offers the potential to address other graphical integrity issues. With this method, we found that graphical integrity issues do appear among scientific articles. Even though the fraction of figures with graphical integrity issues is minor, publishers and research communities might still need to consider taking actions to protect readers from misunderstanding the research findings because of the graphs with graphical integrity issues.

Acknowledgments

HZ, TYH, DEA were partially funded by the ORI HHS grants ORIIR180041 and ORIIIR190049.

References

Acuna, D., Brooks, P., & Kording, K. (2018). *Bioscience-scale automated detection of figure element reuse*.

Al-Zaidy, R. A., & Giles, C. L. (2017, January 1). *A Machine Learning Approach for Semantic Structuring of Scientific Charts in Scholarly Documents*. AAAI. https://openreview.net/forum?id=r1bL5eWOWr

Beattie, V., & Jones, M. J. (1992). The use and abuse of graphs in annual reports: Theoretical framework

and empirical study. *Accounting and Business Research*, *22*(88), 291–303.

Beattie, V., & Jones, M. J. (2002). Measurement distortion of graphs in corporate reports: An experimental study. *Accounting, Auditing & Accountability Journal*.

Beck, T. S. (2016). Shaping Images. In *Shaping Images*. De Gruyter Saur. https://www.degruyter.com/document/doi/10.1515/9783110477092/html

Bergstrom, C. T., & West, J. D. (2020a). *Calling Bullshit: The Art of Skepticism in a Data-Driven World* (Illustrated Edition). Random House.

Bergstrom, C. T., & West, J. D. (2020b). *Calling Bullshit: The Art of Skepticism in a Data-Driven World* (Illustrated Edition). Random House.

Bergstrom, C., & West, J. (2021). *Tools—Proportional Ink and Logarithmic Scales*. https://www.callingbullshit.org/tools/logarithmic_scales.html

Bik, E. M., Casadevall, A., & Fang, F. C. (2016a). The Prevalence of Inappropriate Image Duplication in Biomedical Research Publications. *MBio*, *7*(3), Article 3. https://doi.org/10.1128/mBio.00809-16

Bik, E. M., Casadevall, A., & Fang, F. C. (2016b). The Prevalence of Inappropriate Image Duplication in Biomedical Research Publications. *MBio*, *7*(3). https://doi.org/10.1128/mBio.00809-16

Bochkovskiy, A., Wang, C.-Y., & Liao, H.-Y. M. (2020). YOLOv4: Optimal Speed and Accuracy of Object Detection. *ArXiv:2004.10934 [Cs, Eess]*. http://arxiv.org/abs/2004.10934

Chagas, P., Akiyama, R., Meiguins, A., Santos, C., Saraiva, F., Meiguins, B., & Morais, J. (2018). Evaluation of Convolutional Neural Network Architectures for Chart Image Classification. *2018 International Joint Conference on Neural Networks (IJCNN)*, 1–8. https://doi.org/10.1109/IJCNN.2018.8489315

Choudhury, S. R., Wang, S., & Giles, C. Lee. (2016). Scalable algorithms for scholarly figure mining and semantics. *Proceedings of the International Workshop on Semantic Big Data*, 1–6. https://doi.org/10.1145/2928294.2928305

Clark, C., & Divvala, S. (2016). PDFFigures 2.0: Mining Figures from Research Papers. *Proceedings of the 16th ACM/IEEE-CS on Joint Conference on Digital Libraries*, 143–152. https://doi.org/10.1145/2910896.2910904

Cliche, M., Rosenberg, D., Madeka, D., & Yee, C. (2017). Scatteract: Automated Extraction of Data from Scatter Plots. In M. Ceci, J. Hollmén, L. Todorovski, C. Vens, & S. Džeroski (Eds.), *Machine Learning and Knowledge Discovery in Databases* (pp. 135–150). Springer International Publishing. https://doi.org/10.1007/978-3-319-71249-9_9

Committee on Publication Ethics. (2021). *Core practices | COPE: Committee on Publication Ethics*. https://publicationethics.org/core-practices

Courtis, J. K. (1997). Corporate annual report graphical communication in Hong Kong: Effective or misleading? *The Journal of Business Communication (1973)*, *34*(3), 269–284.

Cromey, D. W. (2010). Avoiding Twisted Pixels: Ethical Guidelines for the Appropriate Use and Manipulation of Scientific Digital Images. *Science and Engineering Ethics*, *16*(4), 639–667. https://doi.org/10.1007/s11948-010-9201-y

Epshtein, B., Ofek, E., & Wexler, Y. (2010). Detecting text in natural scenes with stroke width transform. *2010 IEEE Computer Society Conference on Computer Vision and Pattern Recognition*, 2963–2970. https://doi.org/10.1109/CVPR.2010.5540041

García Seco de Herrera, A., Schaer, R., Bromuri, S., & Müller, H. (2016, September). Overview of the ImageCLEF 2016 medical task. *Working Notes of CLEF 2016 (Cross Language Evaluation Forum)*.

Gonon, F., Bezard, E., & Boraud, T. (2011). Misrepresentation of Neuroscience Data Might Give Rise to Misleading Conclusions in the Media: The Case of Attention Deficit Hyperactivity Disorder. *PLOS ONE*, *6*(1), e14618. https://doi.org/10.1371/journal.pone.0014618

Grudniewicz, A., Moher, D., Cobey, K. D., Bryson, G. L., Cukier, S., Allen, K., Ardern, C., Balcom, L., Barros, T., Berger, M., Ciro, J. B., Cugusi, L., Donaldson, M. R., Egger, M., Graham, I. D., Hodgkinson, M., Khan, K. M., Mabizela, M., Manca, A., … Lalu, M. M. (2019). Predatory journals: No definition, no defence. *Nature*, *576*(7786), 210–212. https://doi.org/10.1038/d41586-019-03759-y

Jordan, S. R. (2014). Research integrity, image manipulation, and anonymizing photographs in visual social science research. *International Journal of Social Research Methodology*, *17*(4), 441–454. https://doi.org/10.1080/13645579.2012.759333

Larkin, A. A. (1990). *Misleading Graphics: Can Decision Makers Be Affected By Their Use*.

Lee, P., West, J. D., & Howe, B. (2018). Viziometrics: Analyzing Visual Information in the Scientific Literature. *IEEE Transactions on Big Data*, *4*(1), 117–129. https://doi.org/10.1109/TBDATA.2017.2689038

Lee, P.-S., West, J. D., & Howe, B. (2018). Viziometrics: Analyzing Visual Information in the Scientific Literature. *IEEE Transactions on Big Data*, *4*(1), 117–129. https://doi.org/10.1109/TBDATA.2017.2689038

Lee, S. L., & Zare, M. R. (2018). Biomedical compound figure detection using deep learning and fusion techniques. *IET Image Processing*, *12*(6), 1031–1037. https://doi.org/10.1049/iet-ipr.2017.0800

Li, P., Jiang, X., & Shatkay, H. (2019). Figure and caption extraction from biomedical documents. *Bioinformatics*, *35*(21), 4381–4388. https://doi.org/10.1093/bioinformatics/btz228

Lo, K., Wang, L. L., Neumann, M., Kinney, R., & Weld, D. S. (2020). S2ORC: The Semantic Scholar Open Research Corpus. *ArXiv:1911.02782 [Cs]*. http://arxiv.org/abs/1911.02782

Luo, J., Li, Z., Wang, J., & Lin, C.-Y. (2021). *ChartOCR: Data Extraction From Charts Images via a Deep Hybrid Framework*. 1917–1925. https://openaccess.thecvf.com/content/WACV2021/html/Luo_ChartOCR_Data_Extraction_From

_Charts_Images_via_a_Deep_Hybrid_WACV_2021_paper.html

Manning, C. D., Raghavan, P., & Schütze, H. (2008). *Introduction to Information Retrieval*. Cambridge University Press. https://doi.org/10.1017/CBO9780511809071

Moher, D., Bouter, L., Kleinert, S., Glasziou, P., Sham, M. H., Barbour, V., Coriat, A.-M., Foeger, N., & Dirnagl, U. (2020). The Hong Kong Principles for assessing researchers: Fostering research integrity. *PLOS Biology*, *18*(7), e3000737. https://doi.org/10.1371/journal.pbio.3000737

Murtaugh, P. A. (2002). Journal quality, effect size, and publication bias in meta-analysis. *Ecology*, *83*(4), 1162–1166.

Nair, R. R., Sankaran, N., Nwogu, I., & Govindaraju, V. (2015). Automated analysis of line plots in documents. *2015 13th International Conference on Document Analysis and Recognition (ICDAR)*, 796–800. https://doi.org/10.1109/ICDAR.2015.7333871

Office of Research Integrity. (2021). *Definition of Research Misconduct | ORI - The Office of Research Integrity*. https://ori.hhs.gov/definition-research-misconduct

PMC Overview. (2021). https://www.ncbi.nlm.nih.gov/pmc/about/intro/

Poco, J., & Heer, J. (2017). Reverse-engineering visualizations: Recovering visual encodings from chart images. *Computer Graphics Forum*, *36*, 353–363.

Prasad, V. S. N., Siddiquie, B., Golbeck, J., & Davis, L. S. (2007). Classifying Computer Generated Charts. *2007 International Workshop on Content-Based Multimedia Indexing*, 85–92. https://doi.org/10.1109/CBMI.2007.385396

Raschke, R. L., & Steinbart, P. J. (2008). Mitigating the Effects of Misleading Graphs on Decisions by Educating Users about the Principles of Graph Design. *Journal of Information Systems*, *22*(2), 23–52. https://doi.org/10.2308/jis.2008.22.2.23

Savva, M., Kong, N., Chhajta, A., Fei-Fei, L., Agrawala, M., & Heer, J. (2011). ReVision: Automated classification, analysis and redesign of chart images. *Proceedings of the 24th Annual ACM Symposium on User Interface Software and Technology - UIST '11*, 393. https://doi.org/10.1145/2047196.2047247

Shen, C., & Björk, B.-C. (2015). 'Predatory' open access: A longitudinal study of article volumes and market characteristics. *BMC Medicine*, *13*(1), 230. https://doi.org/10.1186/s12916-015-0469-2

Shneiderman, B. (2003). The Eyes Have It: A Task by Data Type Taxonomy for Information Visualizations. In B. B. Bederson & B. Shneiderman (Eds.), *The Craft of Information Visualization* (pp. 364–371). Morgan Kaufmann. https://doi.org/10.1016/B978-155860915-0/50046-9

Shukla, S., & Samal, A. (2008). Recognition and quality assessment of data charts in mixed-mode documents. *International Journal of Document Analysis and Recognition (IJDAR)*, *11*(3), 111. https://doi.org/10.1007/s10032-008-0065-5

Siegel, N., Lourie, N., Power, R., & Ammar, W. (2018). Extracting Scientific Figures with Distantly Supervised Neural Networks. *Proceedings of the 18th ACM/IEEE on Joint Conference on Digital Libraries*, 223–232. https://doi.org/10.1145/3197026.3197040

Sinha, A., Shen, Z., Song, Y., Ma, H., Eide, D., Hsu, B.-J. (Paul), & Wang, K. (2015). An Overview of Microsoft Academic Service (MAS) and Applications. *Proceedings of the 24th International Conference on World Wide Web*, 243–246. https://doi.org/10.1145/2740908.2742839

Sox, H. C., & Rennie, D. (2006). Research Misconduct, Retraction, and Cleansing the Medical Literature: Lessons from the Poehlman Case. *Annals of Internal Medicine*, *144*(8), 609–613. https://doi.org/10.7326/0003-4819-144-8-200604180-00123

Sumner, P., Vivian-Griffiths, S., Boivin, J., Williams, A., Venetis, C. A., Davies, A., Ogden, J., Whelan, L., Hughes, B., Dalton, B., Boy, F., & Chambers, C. D. (2014). The association between exaggeration in health related science news and academic press releases: Retrospective observational study. *BMJ*, *349*, g7015. https://doi.org/10.1136/bmj.g7015

Taschwer, M., & Marques, O. (2018). Automatic separation of compound figures in scientific articles. *Multimedia Tools and Applications*, *77*(1), 519–548. https://doi.org/10.1007/s11042-016-4237-x

Taschwer, M., & Marques, O. (2016). Compound Figure Separation Combining Edge and Band Separator Detection. In Q. Tian, N. Sebe, G.-J. Qi, B. Huet, R. Hong, & X. Liu (Eds.), *MultiMedia Modeling* (pp. 162–173). Springer International Publishing. https://doi.org/10.1007/978-3-319-27671-7_14

Titus, S. L., Wells, J. A., & Rhoades, L. J. (2008). Repairing research integrity. *Nature*, *453*(7198), 980–982. https://doi.org/10.1038/453980a

Tufte, E. R. (2001). *The visual display of quantitative information* (Vol. 2). Graphics press Cheshire, CT.

Vassilieva, N., & Fomina, Y. (2013). Text detection in chart images. *Pattern Recognition and Image Analysis*, *23*(1), 139–144. https://doi.org/10.1134/S1054661813010112

West, J. D., & Bergstrom, C. T. (2021). Misinformation in and about science. *Proceedings of the National Academy of Sciences*, *118*(15). https://doi.org/10.1073/pnas.1912444117

Xiang, Z., & Acuna, D. E. (2020). Scientific Image Tampering Detection Based On Noise Inconsistencies: A Method And Datasets. *ArXiv:2001.07799 [Cs, Eess]*. http://arxiv.org/abs/2001.07799

Yang, B. W., Vargas Restrepo, C., Stanley, M. L., & Marsh, E. J. (2021). Truncating Bar Graphs Persistently Misleads Viewers. *Journal of Applied Research in Memory and Cognition*. https://doi.org/10.1016/j.jarmac.2020.10.002
