# Supplementary material for: Graphical integrity issues in open access publications: Detection and patterns of proportional ink violations
Source: PLoS Comput Biol. 2021 Dec 13;17(12):e1009650. doi: 10.1371/journal.pcbi.1009650 (PMC8700024; doi:10.1371/journal.pcbi.1009650)
Supplement: Attachment — Submitted filename: zhuang_huang_acuna_revision2_reponse.pdf [file pcbi.1009650.s010.pdf]

“Graphical integrity issues in open access publications: detection and patterns of proportional ink violations”: response to reviewers

Han Zhuang, Tzu-Yang Huang, and Daniel E. Acuna
School of Information Studies
Syracuse University

Thanks to the editor for managing a swift review process. We also thank the reviewers for their valuable and constructive comments. The feedback has improved the manuscript substantially, especially around clarifying the objective, results, and analyses. We have made the code and data openly available for the reviewers to see. We hope to continue improving the computer code’s quality, but for now it should suffice to address the reviewers’ request. Taken of all these revisions together, we are confident that we have addressed most of the crucial concerns.

Across reviewers, we think there are two major issues raised: the clarity of the description of our method and the discrepancies between the human performance and the algorithmic performance. We have revised our manuscript extensively to address these issues. Below, the comments from reviewers are in purple and our responses are in black.

Reviewer #1:

Thanks for agreeing to review our manuscript. Your comments helped us to clarify the description, and smooth the flow of what we are trying to describe. Below are each of your comments in order.

Comment 1: For readers who are not familiar with the "proportional ink principle" it might be hard to understand what the study was about, by just reading the title, abstract, and introduction. The paper itself only defines proportional ink violation at the bottom of page 11, and I had to look it up online in order to understand the paper. It is not a difficult concept, though, just something that could easily be explained in one or two sentences in the Introduction. Page 2 already more or less gives the definition in 'For example, truncating the y-axis of bar charts ', but there it was not clear that this refers to proportional ink. This would be a perfect spot to introduce the principle of proportional ink.

Response: Thanks for helping us sharpen the definitions in the paper. This request for clarification and better definition was shared by all reviewers. We have added a more precise definition of the “proportional ink principle” in the introduction section (pp. 2), more carefully distinguishing the difference between Tufte and Bergstrom & West. Now our manuscript reads: “*Several general principles for graphs have been proposed to avoid these issues (Tufte, 2001). One of these principles is the principle of proportional ink. It is a specialized rule derived from one of the general graphical integrity principles introduced by (Tufte, 2001) who stated that ‘the representation of numbers, as physically measured on the surface of the graphic itself, should be directly proportional to the numerical quantities represented.’ Further, the derivation of the definition is succinctly presented by (Bergstrom & West, 2020) stating that ‘when a shaded region is used to represent a numerical value, the size (i.e., area) of that shaded region should be directly proportional to the corresponding value.’*”

Comment 2: Did this paper only look at bar graphs? There is a lot of text on classification but I was not sure which types of images were eventually analyzed. It would be very helpful if the Abstract could contain a short definition of proportional ink, and specifically mention that this paper focused on bar charts (assuming this is the case).

Response: Yes, this manuscript focuses on bar graphs, but the method is sufficiently general to be used in other kinds of plots. We have given a short definition of proportional ink in the abstract: "*In this work, we develop a deep learning-based method to accurately measure violations of the proportional ink principle, which states that the size of shaded areas in graphs should be consistent to their corresponding quantities.*"

We have also made a clarification that we only analyze bar charts in the abstract: "*Our results estimate that 5% of bar charts contain proportional ink violations.*"

Comment 3: The paper describes two study sets of images (see e.g. Table 1) but it was unclear how they differed. They are not clearly described in the Methods. Are both sets subsamples from PMC? Figure 2 shows the two sets are nearly identical but it is unclear how they are different. Confusingly, the 50K set is labeled as "human-annotated" in Table 1 while the manuscript appears to describe that these images were processed by automated tools. The authors mention "traditional steps of chart mining" at the bottom of page 4, but this was very vague. Did the authors manually identify the 8K bar charts from the 50K figures or was this done with some automated extraction? The second set "Predictions" is even less well defined. Is this the set that the authors applied their novel analysis tool on? See additional questions about Table 1 below.

Response: We have now clarified the sampling and labeling process in more detail. Both sets of subsamples are from PMC. This is, both the 50K images set and the 250K images set are randomly sampled from PMC figures. The process by which each set goes to labeling or "predictions" is now explained in Figure. 1 of our Supplementary Material.

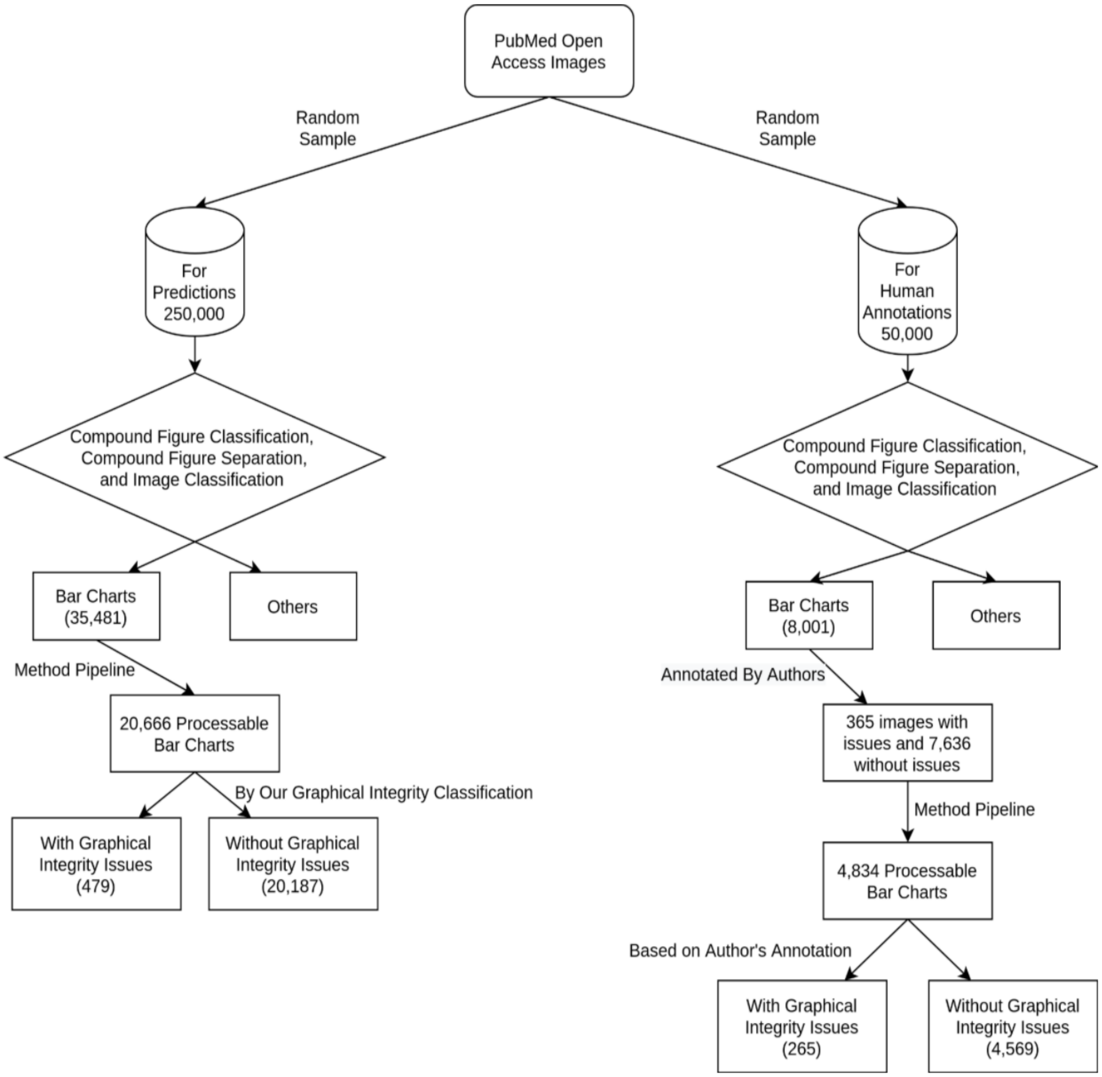

Figure 1. Flowchart of our data source and process. Predictions and Human Annotations data sets are randomly selected from PubMed Open Access Images. Authors annotated 8,001 bar charts from the human-annotated set and 4,834 bar charts could be processed by the method pipeline.

Figure 2 in the main text shows the two sets are similar and they are both random samples from PMC. Thus, we can make inference from the combination of these two sets of images in our analysis.

The reviewer is right that the human-annotated part has a confusing explanation. We have added a more detailed explanation of how the images go through the pipeline for human annotation and for prediction in section 3.1.1.

In the reviewer's question "Did the authors manually identify the 8K bar charts from the 50K figures or was this done with some automated extraction?", we clarify that there is an initial set of 50K images sampled from PMC that are then automatically split into subfigures (i.e., if they are compounded) and then classified into bar charts and other charts. This process generates the 8K bar charts for human

annotation. Finally, the second set of "predictions" is a completely different set of figures whose purpose is to analyze the extent of proportional ink violations. As the reviewer correctly states, this is the set where we applied our analysis tool. We have modified the language in Table 1 and make the headers and comments clarify the nature of the data and annotations.

	Annotation pipeline	Prediction pipeline
Figures	50,000	250,000
Papers**	29,901	68,265
Bar charts	8,001	35,481
Processable bar charts	4,834	20,666
With / Without graphical integrity issues	265 / 4,569	479 / 20,187
Number of papers with processable bar charts	3,094***	10,659

* PMC consists of 7 million papers

***Image (and not the paper) was the unit of sample and analysis*

****In our statistical analysis, we included all human annotated bar charts, instead of processable bar charts*

Table 1. Summary of Annotation pipeline and Prediction pipeline. With annotation pipeline, we applied compound figure classification, subfigure separation, and bar chart classification to obtain bar charts from this sample and then ask annotators to annotate graphical integrity issues on these bar chart. With prediction pipeline, we applied our whole graphical integrity issues detector on this sample. Both sets are similar, as demonstrated by analysis in Fig. 2.

Comment 4: The introduction might be more clear if the authors could mention that Figures in papers can consist of many elements, including photos and line graphs, and that integrity issues can be found in all kinds of image types.

Response: Thanks for the suggestion. We modified the introduction section by adding:

"*Studies driven by human examination have found that common manipulations of graphs and photographic images can have mistakes or integrity violations (Beck, 2016; Bik et al., 2016).*"

Comment 5: The Introduction appears to state that proportional ink violations amount to research misconduct, but that seemed a bit too harsh. I could see someone leaving out the 0 on a bar graph, without the intention to mislead (maybe with good intentions even) but that would not necessarily qualify as research misconduct. There are many sorts of questionable research practices, but it appears too black and white to assume that a violation of proportional ink is always research misconduct. The introduction could benefit from describing the full spectrum of the Perfect Honest Study to questionable research practices to science misconduct.

Response: Thanks for the suggestion. Overall, we have improved how nuanced we are about our language. In the particular case that the reviewer states (i.e., “someone leaving out the 0 on a bar graph”), our method would not predict it as violating the proportional ink principle if the start of the y-axis can be inferred to be close to zero. In other words, the number 0 does not need to be explicitly shown: our method infers the lowest y axis tick based on the rest of the tick labels.

We started off this project with the assumption that most cases are probably OK and we should not assume intentionality in our analysis and predictions. We agree that graphical integrity issues are not necessarily research misconduct. Clearly, our manuscript lacks such a statement. We now state this point earlier and more prominently writing that “*However, assigning intentionality can be highly problematic, requiring expert opinion based on quantitative analysis. Regardless of intentionality, early, accurate, and scalable detection of potential problems during pre and post publication are crucial steps to make science more robust.*”

Comment 6: An additional part of the paper that needs to be better defined is the evaluation of estimating integrity issues. The authors define precision and recall, but they do not define what was compared to what. I assume they compared some automated techniques with some manual assessment, but I could not find this in the paper. Is this done on the 50K or on the 250K set?

Response: We have better defined the evaluation of integrity issues in pp. 5 (section 3.1.2 and section 4.2.4). The precision and recall are based on both our prediction (by our automated method) and our annotation (by human annotator) on the 8,001 bar charts (extracted from the 50K figures, through compound figure detection, subfigure separation, and chart classification). We have rewritten our evaluation process in the 3.1.2 and 4.2.4 sections.

In section 3.1.2 we have added the followings: “*We used Area under the ROC curve (denoted as AUC), precision, and recall with stratified 5-fold cross-validation to evaluate the performance of our graphical integrity evaluator (for our evaluation protocol, see Materials and Methods section 4.2.4).*”

And please see section 4.2.4 in the manuscript for more details.

Comment 7: Maybe I missed it but where is "our algorithm" provided? Is the method given by the authors or described in some reproducible method section? Figure 7 appears too general for others to replicate the method.

Response: The reviewer is right that we do not sufficiently describe in some reproducible manner the “algorithm”. We have decided to change this to “our method” because it really is a combination of algorithms, engineering, and other tools. Also, we have made the method available openly in a GitHub repository (https://github.com/sciosci/graph_check). Accordingly, we have modified the names, captions, and added a link to the code. The reviewer is right about Figure 7 being too general, but it was on purpose. We have clarified this simplification in the caption of Figure. 7. We provide a more detailed explanation of our method in section 4.2.

Comment 8: The authors sometimes claim their new method to determine violation of the proportional ink principle to be highly accurate (e.g. page 16), but on page 8 they write "We found that the percentage of graphical integrity issues is significantly larger in human-annotated than automated prediction bar

charts". This might be my poor understanding of what actually is compared here, but are these two sentences not in conflict with each other?

Response: Our method has a good overall performance. It has a high *Area Under the ROC* curve (called AUC, see our response to Comment 19 for more details) of 0.917 ± 0.02, which is a somewhat technical way of saying that it can successfully sort cases in classification tasks. We have clarified how AUC relates to accuracy in section 3.1.2 (pp. 6). What we were trying to say with the quoted passage was that our method has still room for improvement compared to human annotators. Comparison of algorithms and human annotators can sometimes be confusing as we can change how sensitive our method is, effectively increasing the predicted prevalence and bringing it closer to human annotator prevalence. If we make our method more sensitive, we will be able to increase our *recall* (how often the true graphical integrity issues are detected by our method). However, this increase sacrifices *precision* (how often our predictions are truly correct). We have added a section 3.2.8 called "Error Analysis" doing this kind of sensitivity analysis:

3.2.8. Error Analysis

Interpreting the differences between human annotation and our method improve the transparency of our method but is difficult. To interpret this difference, we conducted a systematic evaluation of how the parameters on our model change the precision, recall, and predicted prevalence in the datasets. This is because we want to examine why our annotator find 5.5% of bar charts having proportional ink violation but our method finds 2.3% of bar charts having proportional ink violation (see 3.2.2 section for more details. We found this discrepancy between predicted *prevalence* and the human-annotated prevalence can the result of the threshold of classifying a bar chart into proportional ink violation in our classifier (see section 4.2.3 for more details). We can reduce the discrepancy between predicted prevalence and the human-annotated prevalence by lowering this threshold. Although this comes at the cost of increasing the false positive rate, we can approximate the human and predicted prevalence rate with a reasonable cost. To be concrete, we explore parameters that would approximate human's prevalence. When we change the default threshold of classifying bar charts to graphical integrity issues from 0.5 to 0.23, we can improve our predicted prevalence rate of graphical integrity issues from 2.3% to 3.4%, without losing *F1* score (see Figure 4 in the Supplementary Material). Thus, depending on the ultimate goals of the system, we do not necessarily want to approximate human prevalence rate in this fashion. We believe that the choice of such threshold should be in the hands of the users of the systems—ultimately, authors, journals, and universities.

We also performed a systematic analysis of the false positive and false negative classifications to determine whether we need more data, more sophisticated methods, or better feature engineering (see examples in Supplementary Material section 2.2). The overall takeaway from this analysis is that our method has issues detecting bar charts with a partially hidden y-axis. This is because for our method, we need highly accurate text localization and text recognition on y-axis to perform well on the detection of these violations. However, methods for text localization are an area of active research (Chen et al., 2021).

Comment 9: Page 1, Abstract. Suggested rewording: "However, the extent of violations of these principles in academic publications is unknown."

Response: Thank you for the suggestion. We have made the change

Comment 10: Page 1, Introduction. "Intentionally or unintentionally". Isn't research misconduct not usually done intentionally?

Response: Thanks for your comment. We have revised the sentence to

"However, assigning intentionality can be highly problematic, requiring expert opinion based on quantitative analysis."

Comment 11: Page 1. 'A survey of researchers revealed': the authors cite an older study. Are there any more recent papers? Two recent preprints on this topic could be added: https://osf.io/preprints/metaarxiv/vk9yt/ and https://osf.io/preprints/metaarxiv/xsn94/

Response: Thanks for these suggestions. These two articles are more recent, and we have cited the survey of research misconduct in the introduction:

"Surveys of researchers have revealed that from 4% to 9% of scientists have observed or have evidence of others' research misconduct (Gopalakrishna et al., 2021; Titus et al., 2008)."

Comment 12: Page 2, 'For example, truncating the y-axis of bar charts ' would be a perfect spot to introduce the principle of proportional ink.

Response: We have added the definition of the principle of proportional ink in page 2. We added the following -

"Several general principles for graphs have been proposed to avoid these issues (Tufte, 2001). One of these principles is the principle of proportional ink. It is a specialized rule derived from one of the general graphical integrity principles introduced by (Tufte, 2001) who stated that 'the representation of numbers, as physically measured on the surface of the graphic itself, should be directly proportional to the numerical quantities represented.' Further, the derivation of the definition is succinctly presented by (Bergstrom & West, 2020) stating that 'when a shaded region is used to represent a numerical value, the size (i.e., area) of that shaded region should be directly proportional to the corresponding value.'"

Comment 13: Page 2. 'For example, researchers have developed computer techniques to detect image tampering and image reuse' - this is focusing more on photographic images, correct? Or were graphs also included? Might need to clarify here that these were done on photos, and that this paper will look at graphs.

Response: We have revised the sentence to

"Researchers have proposed automated and scalable techniques to detect violations of research integrity. The present study looks at another kind of scientific imagery that has been somewhat less explored before: graphs."

Comment 14: Page 2. 'These studies reveal the prevalence of image fabrications in science beyond small samples of images (Bik et al., 2016a).' That study was a manual analysis of photographic images, not an automated technique applied to all image types.

Response: We modified the sentence to

"Studies driven by human examination have found that common manipulations of graphs and photographic images can have mistakes or integrity violations (Beck, 2016; Bik et al., 2016)."

Comment 15: Page 3: 'Data extraction. It is stated that 'charts are images without data' but the text then continues to extract the data from the images. This was a bit confusing; charts contain a lot of data. Do the authors mean that those graphs do not contain actual numbers?

Response: Sorry about the confusion. We change the sentence to

" *A bar chart, plot, and pie chart panels might contain valuable information to be extracted from the image such as point locations, tick values, and statistical information.*"

Comment 16: Page 4. Chapter 2.3. Typo. Title of paragraph should be "Chart mining". Also, check sentence 'significant challenges left when deadline with low resolution images' - should this be 'dealing'?

Response: We have corrected the spelling to "*chart mining*" and the spelling from "deadline" to "dealing".

Comment 17: Page 4. Chapter 2.2 "Datasets about science". Should this not be 2.4? Also, 'In the recent past, we lacked the datasets necessary to analyze science.' - do the authors mean 'to analyze the scientific literature'?

Response: We have corrected the index to 2.2.1. Yes, by "analyze science", we are referring to entities in science, such as full texts, images and data in publications. Now, our manuscript reads: "*Chart mining techniques benefit from large datasets. Only recently, we have access to a much broader set of information pieces about science than before.*"

Comment 18: Page 4, Chapter 3.1.1/Table 1. It might be helpful to refer to Table 1 here. What is meant by "We labeled images" - do the authors mean "we analyzed"? And by "images" do the authors mean figures or figure panels? What is meant by "we perform the traditional steps of chart mining"? Is this a manual screen of the 50,000 figures, as suggested by "human annotations" in Table 1? Or were these images extracted with some automated tool? This was very confusing.

Response: Thanks for the feedback, and sorry about the confusion. Yes, we analyzed the image sample for our annotation pipeline. By "images", we refer to non-compound figures and subfigures. To clarify, we have changed the term "the traditional steps of chart mining" to "*We then perform compound figure separation, and bar chart detection (i.e., figure classification) (see Fig. 1).*". Reviewer asked: "Is this a manual screen of 50,000 figures?" No, we did not conduct a fully manual screen of 50,000 figures. We processed 50,000 figures with our automated methods to get bar charts and then annotated the 8001 bar charts that were detected (previously called human annotations in Table 1).

This is the new Table 1:

	Annotation pipeline	Prediction pipeline
Figures	50,000	250,000
Papers**	29,901	68,265
Bar charts	8,001	35,481
Processable bar charts	4,834	20,666
With / Without graphical integrity issues	265 / 4,569	479 / 20,187
Number of papers with processable bar charts	3,094***	10,659

* PMC consists of 7 million papers

***Image (and not the paper) was the unit of sample and analysis*

****In our statistical analysis, we included all human annotated bar charts, instead of processable bar charts*

Table 1. Summary of Annotation pipeline and Prediction pipeline. With annotation pipeline, we applied compound figure classification, subfigure separation, and bar chart classification to obtain bar charts from this sample and then ask annotators to annotate graphical integrity issues on these bar chart. With prediction pipeline, we applied our whole graphical integrity issues detector on this sample. Both sets are similar, as demonstrated by analysis in Fig. 2.

Comment 19: Page 5, Chapter 3.1.2. I did not understand the AUC as a performance measure. How did the authors determine true and false positivity? What is the gold standard here? Is that done on the "Predictions" Dataset? Against what is this measured? Is this a comparison of a manual analysis vs a computer analysis? I was completely lost here.

Response: As the reviewer can see in the previous comments and our overall response, we have attempted to describe our evaluation much more carefully and reproducibly in our revision. We now first describe what the AUC is and then what we consider true and false positivity.

The AUC is a performance measure that is meant to quantify how well a binary classifier works on unbalanced datasets (e.g., the great majority of graphs does not have graphical integrity violations). Other measures such as accuracy, precision, and recall do not do this well. In terms of true and false positive, the AUC curve represents all possible combinations of true positive and false positive rate as we systematically change the sensitivity of our method. We have added definition to true positive in section 4.2.4: "*Before we introduce how to interpret AUC, we first explain how we define true data points, false data points, positive data points, and negative data points, true positive data points, false positive data points, true negative data points and false negative data points. Here, true data points are images, which have an annotation as a proportional ink violation. False data points are images, which have an annotation as no proportional ink violation. Positive data points are images, which have a prediction from our method as a proportional ink violation. Negative data points are images, which have a*

prediction from our method as no proportional ink violation. True positive data points are images, which have a prediction from our method as a proportional ink violation and also have an annotation as a proportional ink violation. False positive data points have a prediction from our method as a proportional ink violation but have an annotation as no proportional ink violation. True negative data points are images, which have a prediction from our method as no proportional ink violation and an annotation as no proportional ink violation. In addition, false negative data points are images, which have a prediction from our method as no proportional ink violation but have an annotation of proportional ink violation."

The gold standard to determine true and false positive comes from human annotators who went through the systematic evaluation of 8K bar charts. The reviewer is right that during the "prediction" dataset, we do not really have a gold standard. Our reported AUC and other measures of performance are not for this "prediction" dataset. However, we are assuming that the performance of our method would generalize to the "prediction" dataset because in principle we sample both datasets exactly the same. This conclusion is backed up by our analysis of the features used in both datasets: the feature sets are nearly indistinguishable (section 3.1.3). We have expanded our explanation of this difference in section 3.1.2.

In section 4.2.4, we explain precision and recall:"*We additionally investigated the precision and recall of our method. Precision is computed as Eq. (1), which is the ratio of the number of true positive data points to the number of all positive data points (Manning et al., 2008). The recall is the ratio of the number of true positive data points to the number of all true data points (Eq. (2)).*"

Comment 20: Page 8. Chapter 3.2.2. 'Our algorithm' - not defined. Is that one that can recognize and extract bar charts starting with a large amount of PMC images or from pdfs? Or is it an algorithm that works on human-selected bar graphs to determine if e.g. the y axis starts with 0 or not? How did a human extract bar graphs then? Typo: "extend".

Response: We have corrected the typo "extend". Also, we have changed "our algorithm" to "our method" and refer to our method section (4.2). Our method analyzes PMC images, instead of PDFs. We have significantly expanded the explanation of our method in section 4.2. Please also see our response to Comment 7.

Comment 21: Page 8, section 3.2.3. Check sentence: 'higher the ranking (here, "higher" means closer to 1), the more likely to have articles with graphical integrity issues'

Response: Thank you. We modified the sentence to

"*We tested the correlation between the rank of journals and journals' likelihood of having at least one article with graphical integrity issues. To measure this likelihood, we first compute the maximum likelihood that a publication contains a graphical integrity issue. Then, we compute the median of this value per journal. For the journal rank, we use the rank based on the importance of papers in the network of publications from Microsoft Academic Graph (DarrinEide, 2021). For example, a highly cited paper in a prestigious journal has more importance than a preprint article. A Pearson correlational analysis revealed that there is no correlation that the higher the ranking (r = -0.01, N = 1357, p-value = 0.63, Fig. 3).*"

Comment 22: Page 9, figure 3 - I had trouble interpreting this figure. Do the dots at the top of the graph mean that 100% of papers in certain journals have proportional ink issues? Also, the linear regression

line, in particular the rise on the left hand side, appears to be a bit of a stretch. Does that mean that high impact journals have a higher chance of including articles with graphical integrity issues? That is different than the hypothesis stated in 3.2.3. Also typo in figure: "articles'. The title of the graph also appears to be very general " graphical integrity issues" could be more specific, i.e. proportional integrity issues with bar charts. How many papers/images were included in this graph?

Response: Sorry for the confusion. 100% means that the likelihood of proportional ink violations in a sample of images for a journal is 100% because human annotators assign violation (100%) or not (0%) to each image and a sample of images for a journal can be all violations. However, this assumption is not always correct.

To further address this confusion in our manuscript, we only analyze figures from the prediction pipeline, which do not have a probability of 100%. There are 20,204 figures and we aggregated the likelihood of these figures to the paper level (10,143 papers).

We have revised the title of figures 3,4,5, and 6 to "*likelihood of having bar charts violating principle of proportional ink in an article*".

Comment 23: Page 9, figure 4. How many papers/images were included in this graph?

Response: That graph includes 10,143 papers and 20,204 images. In this graph, we present the proportional ink violations in papers across fields, so we aggregate the likelihood of proportional ink violation of images into the likelihood of proportional ink violation of papers by taking the maximum likelihood of proportional ink violation of images in each paper.

Comment 24: Page 10, Chapter 3.2.5. How did the authors determine the country of a paper? Did they include all affiliations, or only those of the corresponding author?

Response: We identify the country of authors by their affiliations. We have also corrected the index of sections to 3.2.5. Yes, our data has affiliations of all authors, instead of the corresponding author. We have also explained this on page 9:

"*Here, we examined the likelihood of having articles with graphical integrity issues for all their authors from the country of their affiliations.*"

Comment 25: Page 11, section 4.1.1. 'mentioned in the previous subsection' - should this not be 'mentioned in the following subsection'?

Response: Sorry for the confusion, we change it to "*mentioned in section 3.1.1.*"

Comment 26: Page 12, Chapter 4.1.3. "See the summary of training data and validation data in 2" - should this be Figure 2? Table 2?

Response: Sorry for the mistake. We have added "Table. 2" to the sentence

Comment 27: Page 12, Impact of journals. How was the journal rank determined? I imagine there are multiple indexes to calculate this - which one was used? Or was impact factor used to determine rank?

Response: The journal rank is determined by the likelihood of being "important" in the network of publications (DarrinEide, 2021). This importance is measured through a paper's relationship with other papers in the citation network.. For example, a highly cited paper in a prestigious journal is more important than a preprint article. The Microsoft Academic Graph is scarce in the details about how they compute this likelihood and describe the formula as:
$Rank = -1000 \times Ln\,(probability\ of\ an\ entity\ being\ important)$ .We expand our explanation of impact of journals as

"*For the journal rank, we use the rank based on the importance of papers in the network of publications from Microsoft Academic Graph (DarrinEide, 2021). For example, a highly cited paper in a prestigious journal has more importance than a preprint article.*"

to page 12.

Comment 28: Page 13, Research fields. "We collected the field of study of journals of articles in our sample." - how was this determined? Which fields from which databases were used? Or was this manually determined? More details are needed here.

Response: The research field is automatically determined by a multi-class classification technique, based on publications' content and their relationship with other publications. For all our metadata, we use the Microsoft Academic Graph (MAG). MAG manually defines a pool of fields or categories and conducts a multi-class prediction on the fields of publications. We have added "*The research field of journals is selected by researchers from Microsoft Academic Graph and each publication is classified into research fields by a multi-class classification method with publications' content and connections in the network of publications.*" to section 3.2.4

Comment 29: Page 13 'After we have compound figures' --> 'In the case of a compound figure'

Response: We have changed the sentence to "*In the case of a compound figure*"

Comment 30: References. Some papers appear to be included twice. Examples: Bergstrom C et al. 2020a/2020b; Bik et al. 2016a/2016b; Lee P 2018.

Response: We have removed duplicated references

Reviewer #2:

Thanks for agreeing to review this manuscript. Your comments really pushed us to clarify the errors that our method was making. Also, we realize that it would be pertinent to share the code at this stage—even if it is not the final quality we want once the paper is public. We address each of your comments in order below:

Comment 1: The authors report graphical integrity issues in 6.5% of the publications with bar charts and 5.5% per bar chart when hand labeling. They then see a drop after the automated detection (3.6% and 2.3% respectively). What might explain this drop? What could be done with the methods to improve this? The authors noted this underestimation, but I think it would improve the integrity of the results if this was further investigated. I wonder if there is something fairly straightforward that could be addressed that

would either explain the gap or reduce the gap. Since the results are cut nearly 50% when automating, it indicates a potentially serious issue when moving to automation. This needs to be addressed in order to improve confidence in automated detection.

Response: Thanks for pointing out this apparent contradiction. There are two issues: one is a gap in the performance of our method, as suggested by the reviewer, and the other is the evaluation method we used to measure the method's performance. We have added a new section 3.1.3 "Error analysis" detailing both of these issues (please see response to comment #8 for reviewer #1 too).

Regarding the gap in performance, we have investigated the false negatives and false positives. The main errors that our method is making is that bar charts with unusually large truncated y-axes need more accurate automated labels. This large truncation in effect translates to a large jump in y-axis values that confuses the OCR (Optical Character Recognition) method and ultimately our method. Also, our method has challenges with bar charts with low image quality (e.g., too compressed). We suggest a number of improvements for future work, including parsing the images directly from the PDFs instead of the preprocessed images provided by Pubmed Open Access Subset, which seem to have lower quality. In the paragraph of future work, we added: "*Our future work would benefit from higher quality images. According to our error analysis, a large proportion of our method's mistakes is due to low quality images. PubMed Open Access subset makes the article's figures available as separate images, but they are sometimes of lower quality compared to those inside the PDFs. Therefore, future work will attempt to extract images directly from PDFs. Lastly, improvements in these image analysis steps are an active area of research, and therefore should be improved in the future.*"

Regarding the issue with the choice of how we evaluate our method, we have thought deeply about how changes to our method's decision threshold may produce the discrepancy between predicted prevalence (i.e., 2.3%) vs. the human annotator prevalence (i.e., 5.5%). In a few words, our method can produce a range of predicted prevalence depending on how sensitive we make it for detecting a graphical integrity violation. By definition, if we lower the detection threshold, we increase the predicted prevalence rate at the expense of how precise (true positive divided by true position + false positive) our method is. After the reviewer's comments and the error analysis, we have decided to lower the threshold to bring our predicted prevalence closer to human annotation while trying to maintain a good precision. We have devoted a section to this explanation and added:

3.2.8. Error Analysis

Interpreting the differences between human annotation and our method improve the transparency of our method but is difficult. To interpret this difference, we conducted a systematic evaluation of how the parameters on our model change the precision, recall, and predicted prevalence in the datasets. This is because we want to examine why our annotator find 5.5% of bar charts having proportional ink violation but our method finds 2.3% of bar charts having proportional ink violation (see 3.2.2 section for more details. We found this discrepancy between predicted *prevalence* and the human-annotated prevalence can the result of the threshold of classifying a bar chart into proportional ink violation in our classifier (see section 4.2.3 for more details). We can reduce the discrepancy between predicted prevalence and the human-annotated prevalence by lowering this threshold. Although this comes at the cost of increasing the false positive rate, we can approximate the human and predicted prevalence rate with a reasonable cost. To be concrete, we explore parameters that would approximate human's prevalence. When we change the default threshold of classifying bar charts to graphical integrity issues from 0.5 to 0.23, we can improve our predicted prevalence rate of graphical integrity issues from 2.3% to 3.4%, without losing *F1* score (see Figure 4 in the Supplementary Material). Thus, depending on the ultimate goals of the system, we do not necessarily want to approximate human prevalence rate in this fashion. We believe that the choice of such threshold should be in the hands of the users of the systems—ultimately, authors, journals, and universities.

We also performed a systematic analysis of the false positive and false negative classifications to determine whether we need more data, more sophisticated methods, or better feature engineering (see examples in Supplementary Material section 2.2). The overall takeaway from this analysis is that our method has issues detecting bar charts with a partially hidden y-axis. This is because for our method, we need highly accurate text localization and text recognition on y-axis to perform well on the detection of these violations. However, methods for text localization are an area of active research (Chen et al., 2021).

Comment 2: The core contribution relates to the principle of proportional ink. This was a term that the calling bullshit project wrote about in 2017 [Bergstrom, Carl, and Jevin West. "The Principle of Proportional Ink." URL: https://callingbullshit. org/tools/tools proportional ink. html (2017)]. The authors cite Tufte, which is correct for the more general principle of data ink, but Tufte did not come up with the principle of proportional ink. The authors cite Tufte in the first paragraph of their literature review in their description of the principle. Although the principle of proportional ink is inspired by Tufte's, it is a special case of his more general principle that Tufte talks about.

Response: Thanks for pointing out this confusion. We have now correctly attributed Proportional Ink Principle to Bergstrom and West's work in the introduction and Graphical Integrity to Tufte:

"*Several general principles for graphs have been proposed to avoid these issues (Tufte, 2001). One of these principles is the principle of proportional ink. It is a specialized rule derived from one of the general graphical integrity principles introduced by (Tufte, 2001) who stated that 'the representation of numbers, as physically measured on the surface of the graphic itself, should be directly proportional to the numerical quantities represented.' Further, the derivation of the definition is succinctly presented by (Bergstrom & West, 2020) stating that 'when a shaded region is used to represent a numerical value, the size (i.e., area) of that shaded region should be directly proportional to the corresponding value.'*"

Comment 3: More follow-up on the interpretation of the results would further strengthen the paper. For example, how would the results differ from the non-open access literature?

Response: We agree completely. We now discuss these potential differences between open vs. non-open access literature. We found that non-open access literature can be more highly ranked journals or are from high-impact researchers because some fields might have fewer high prestigious open access journals (Swan & Brown, 2004). We do not think we have enough evidence to draw a conclusion about non-open access journals because we do not analyze them.

We added the following to our discussion section: "However, our result might not be generalizable to non-open access journals. This is because in some research fields, their non-open access journals have a higher impact than open access journals (Swan & Brown, 2004) and can have a different review process from open access journals. Thus, the sample of figures from open access journals and the sample of figures from non-open access journals might not be similar enough for us to infer the prevalence of proportional ink violations from open access journals to non-open access journals"

Comment 4: What are aspects of the bar charts could be examined other than just the Y-axis (e.g., width of bar chart)?

Response: There are several aspects of the bar charts to be examined. For example, measuring distortion is another aspect we can examine (Beattie & Jones, 2002). We have added a description of future work on more diverse aspects of graphical integrity on pp. 21: "*In the future, we hope to incorporate this analysis with other kinds of graph checks. For example, we can study image reuse and tampering at different units of figures (compound figure or subfigure) and in a specific kind of figures (Radiology figures or Microscope figures) (Acuna et al., 2018; Xiang & Acuna, 2020). Beyond graphical integrity issues, our results could be part of other kinds of chart analysis, such as the aspect of distortion (Beattie & Jones, 2002) and clearness (Jambor et al., 2021). For example, our method can be a part of studies of the quality of visual communications, from the perspective of human perception and learning (Shukla & Samal, 2008).*"

Comment 5: When reporting results, how much to the violations break the rule — not just whether they break it or not? There are likely some violations that are minor versus ones that are egregious.

Response: We agree that some violations break the rule to a larger extent, but some might not. To fully consider the extent of the violation, we need to consider the context of bar charts, and this might need assistance from domain experts. Based on the information from graphs only, our method can return a higher probability of having graphical integrity issues with a bigger value of the lowest y-axis label. For example, if we keep other features the same, our method generally gives a higher probability to a bar chart with a bigger value of the lowest y-axis label than zero. Thus, the probability returned from our method can partially reflect the extent of the violation against the rule. There is one metric defined by (Tufte, 2001) to measure accuracy of graphical presentation: Lie Factor. However, to compute the Lie Factor, we need the exact value corresponding to the bars in bar charts, but they are not always available. We discuss this for future work.

Comment 6: What explains some of the country-wide and journal results?

Response: Previous research has found that European countries have their own definitions about research integrity (Desmond & Dierickx, 2021). This diversity of definitions might affect what is considered good

information visualization practices. However, we feel that this is too strong of an explanation to put it in the main text. We hope that future work can address this important issue.

Comment 7: Why is computer science possibly the worst in these violations? Is something about their presentation of results?

Response: We are equally puzzled by this result. Our best guess is that computer science is not a discipline with a tradition in investigating research integrity issues. The principle of proportional ink is a general rule for graphical design in research because it is proposed according to the human vision system (Bertin, 2011; Ware, 2010). The difference of proportional ink violation across research fields can be the result of differences in intensity of investigations and regulations (Haven et al., 2019). In the future, we will investigate this in more detail.

Comment 7: If authors are not repeating errors, what does this mean?

Response: With this topic, we wanted to explore whether being trained or interacting with authors that have integrity issues is predictive of future integrity issues. This phenomenon is what we call "repeating errors". We expand our text to clarify this term in section 3.2.8. It reads "*We now examined whether having experienced graphical integrity issues in the past is predictive of graphical integrity issues in the future. First, we examine if the graphs produced by authors who had graphical integrity issues in the past are more likely to have graphical integrity issues, than the graphs produced by authors who were not found with graphical integrity issues.*"

Comment 8: In the conclusion, the authors mention that a minor problem. But is it minor? 1 out of every 20 articles has a problem. That may be more of a problem (or maybe not). A more thorough discussion here would add to the richness of the study.

Response: We have rewritten it as

"*Even though the fraction of bar charts having proportional ink violations is around 5%, one of these violations might cause some readers to misunderstand the research finding because open access journals are easy to access to a wide range of readers. These misunderstandings might harm readers extensively when the research finding is related to safety or health. With these potential consequences, we suggest publishers and research communities to consider taking actions to protect readers from graphical integrity issues in publications. In the future, we hope to incorporate this analysis with other kinds of graph checks and improve the accuracy of the components in our method.*"

Comment 9: A super interesting result is that higher prestige journals have relatively higher violation rates. Why is that?

Response: We were somewhat puzzled by this result and therefore we re-did the analysis and most of the correlation went away. In our first submission, we aggregated the probability of having proportional ink violation of figures to the likelihood of having proportional ink violation of journals by taking the max probability of having proportional ink violation of figures for each journal. However, some highly ranked journals have more publications and thus more figures in our sample. Considering this fact, we aggregated the probability of having proportional ink violation of figures to paper level by assigning the max likelihood of having proportional ink violation of multiple figures to their paper. Then we aggregate the

likelihood of having proportional ink violation of papers to journals by taking the median likelihood of having proportional ink violation of papers to their journal (see section 3.2.3 for more detail).

Comment 10: I know that the authors don't imply that these errors are solely a result fabrication and purposeful manipulation. I imagine that most of these are honest mistakes and the authors note this in the third sentence of the paper with "intentionally or unintentionally" but then continue the sentence with "fabrication, falsification, or plagiarism." Each of those seems to have an intention to deceive. I recommend rewording to better distinguish the unintentional mistakes and the intestinal falsifications. I just don't want this method to be confused with the ability to detect intention.

Response: We have rewritten the language in the second sentence in the introduction to

"*While inaccuracies can be the result of honest mistakes, some are also research integrity matters (Office of Research Integrity, 2021).*" Please see response to Comment #5 by Reviewer #1 for complementary regarding our lack of evidence to attribute intentionality..

Comment 11: There is potential for publishers to use these methods. This would be a good first line of defense for detecting these kinds of problems before papers are published, but what are some things that publishers should consider if they were to use these methods? Given the policy implications of this work, it would be helpful to go in more depth on the implications of this work and the ways in which the methods could be misused. If done well, it improves the chances of this work to be put in to practice. I don't think this requires a whole new paper but possibly a section devoted to this.

Response: This is an excellent suggestion which we had in mind but perhaps did not come across so clearly in our write-up. We believe many of these types of automated checks could be part of pre-filtering processes during publication submission. The reviewer is right that if publishers use our method, it could be misused. For example, people could actively modify their plots until it is undetectable by our method. We will discuss the implications of this "gaming" process, which does not just apply to our method, but to many others such as image reuse and text plagiarism. We have added the following to our discussion section (pp.19) : "*This work can be a contribution to publishers also. This is because our work shows the possibilities to use AI to help publishers check graphical integrity of scientific articles. Given the overwhelming volume of new manuscript submissions, editors and reviewers face the challenges of maintaining the quality of publications. One potential way of reducing the workload of editors and reviewers is to apply AI to conduct some basic checks on manuscripts. With such considerations, one utility of this method is to analyze a large collection of graphs to detect the violations of the principle of proportional ink. Also, our method provides compound figure classification, subfigure separation, chart classification for publishers to investigate graphical integrity, image reuse or image fabrications. Publishers could use these components extracted from the figures for other analyses.*"

Comment 12: The presentation needs improvement. For example, the abstract would be improved with more specific statements of the results. The authors state that their "results reveal that graphical integrity issues are significantly more prevalent in some types of journals, research fields, and regions of the globe."

Response: We have substantially revised our style and flow in line with the other reviewers' comments. In particular, we revised the abstract to add

"*Our results estimate that 5% of bar charts contain proportional ink violations. Further analysis reveals that these graphical integrity issues are significantly more prevalent in some research fields, such as psychology and computer science, and some regions of the globe, such as Europe.*"

In section 5, We also explore explanations as to why some areas of the globe and research field have this pattern.

Comment 13: The literature review is sparse and disconnected into a series of bullet-point like lists.The literature review is sparse and disconnected into a series of bullet-point like lists. Section 2.2, which is an important part of the paper, is presented more as a series of short notes rather than an integrated discussion of the various methods for chart mining. There are missed follow-ups. For example, the authors note that "it is hard to detect and has detrimental effects." What are the detrimental effects? In presenting the general idea, it would be helpful to provide some specific examples of charts that violate this principle and how it impacts the interpretation of the results.

Response: Thank you for your feedback on our literature review. We have now synthesized our work into a more coherent intellectual piece rather than a bullet-point like list. We have clarified the challenges and potential solutions to each of the points in Section 2.2. In particular, when we mentioned "detrimental effects", we were thinking about the changes that the graph produce on readers' decisions and have further explained what such influences can be in empirical studies.

We also have rewritten the literature review section and have added a couple of examples of violations of the principle of proportional ink to Figure 2 in our supplementary material.

Comment 14: The section 3.1.2 is important for explaining to a more general audience how the results were analyzed. I found the section sparse and difficult to follow. In particular, it is critical to helping the reader interpret results like "0.77 with 0.0209 standard error." What does this mean for a general reader? Is this good, bad or dependent on other aspects of the problem and results? There are multiple listings of references in the bibliography.

Response: Thanks for this comment. We have significantly expanded our explanation of the evaluation system. Please see our response to comment #6 by reviewer #1. In particular, we have rewritten section 3.1.2 to add the following "*In addition, the average precision across folds is 0.77 with 0.0209 standard error. This result shows that around 77% of our positive predictions are true positive violations.*" And "*In sum, these results reveal our method has its strength in making sure positive predictions are true while being relatively less successful with false negative predictions (see 3.1.4 error analysis for more detail).*" In our error analysis section (3.2.8), we expanded this discussion for readers.

Comment 15: "estimating the extent of graphical integrity issues"

Response: We have corrected the typo to "*extent*"

Comment 16: "In the future, we hope to incorporate this analysis with other kinds of integrity checks such as"

Response: We have added the following paragraph about the future work to continue our research about graphical integrity on page 21.

"*In the future, we hope to incorporate this analysis with other kinds of graph checks. For example, we can study image reuse and tampering at different units of figures (compound figure or subfigure) and in a specific kind of figures (Radiology figures or Microscope figures) (Acuna et al., 2018; Xiang & Acuna, 2020). Beyond graphical integrity issues, our results could be part of other kinds of chart analysis, such as aspect of distortion (Beattie & Jones, 2002) and clearness (Jambor et al., 2021). For example, our method can be a part of studies of the quality of visual communications, from the perspective of human perception and learning (Shukla & Samal, 2008).*"

Comment 17: "Figure 2 title: "therefore validating our prediction step"

Response: We have changed the title to "*Features of both datasets are nearly identical. The prediction dataset could provide a reasonable estimation of the more general problems of graphical integrity.*"

Comment 18: "We then analyzed at the author level." Weren't you already doing this?

Response: We were doing the analysis at the chart level, and we explained in section 3.2.8 as "*First, we examine if the graphs produced by authors who had graphical integrity issues in the past are more likely to have graphical integrity issues, than the graphs produced by authors who were not found with graphical integrity issues.*" Then we aggregated charts into authors and conducted the analysis at the author level.

Comment 19: One of the key contributions of this paper is the data set of hand labeled bar charts and automated labeling results and the code for extracting and automating the detection of this rule. They authors note that the data and code will be made available in the PLoS front files, but I did not see it in the main manuscript and could not find the GitHub links. This would be useful for reviewing the methods and results, but at the very least, I hope this is made available upon publication.

Response: We have created a code repository and a data repository. The link to our code repository: https://github.com/sciosci/graph_check. The link to our data repository: https://zenodo.org/record/5500684#.YTvYclspBhF. They will be disseminated upon publication, but for now only the reviewers know that they exist.

Reviewer #3:

Thanks for agreeing to read and review this manuscript. We address each of your comments in order, but we would invite reviewer #3 to also look at our responses to reviewer #2.

Comment 1: you address Tufte in the literature review, but not theorists who address aspects of the perception of visual information, such as Bertin, or Ware. At the same time, in 4.2.2 feature 3, you name the distance of the y-axis label from the x-axis as a possible factor that can undermine integrity-isn't that also a perceptual factor? What is your assessment of the importance of perceptual factors?

Response: Thanks for this comment. Perception is very much at the core of the presentation of visual information, and citing that literature was certainly an overlook from our side. We have now incorporated Bertin and Ware's work to our literature review section:

“*From the perspective of visual perception theory, the principle of proportional ink is based on how humans perceive the size of symbols in graphs (Bertin, 2011) and the ratio of entities in graphs (Ware, 2004).*”

For feature 3, we are not intended to use it as a perceptual factor. We name feature 3 “the distance of the y-axis label from the x-axis” is to estimate how likely we missed the lowest y-axis label in our text localization and text recognition. If this distance is long, we will have to infer the value of the lowest y-axis label for our further analysis because the lowest y-axis label should not be too far from the x-axis.

For the importance of perceptual factors, they are essential to our method and the foundations of the principle of proportional ink because the principle of proportional ink is according to the human perceptual system. With the principle of proportional ink, we can use just basic information on bar charts to detect proportional ink violation, instead of using perceptual factors in graphs directly.

Comment 2: You describe the results of the analysis and a conservative estimate of the number of inappropriate graphs in the database in section 3.2.2. It remains unclear though how high the proportion of publications with graph integrity problems is compared to the total of all analyzed publications. This is certainly because you focused exclusively on publications with bar charts, but it would be helpful for readers to get an idea of the extent of the problem.

Response: Thanks for this suggestion. We have highlighted our scope of analysis as violations of principle of proportional ink in bar charts in section 3.2.1 (pp. 7):

“*Violation of principle of proportional ink is one typical kind of graphical integrity issues.*”

“*In our human annotated dataset, we found 265 proportional ink violations in the 4,834 annotated bar charts that could be processed (6.5 % (± 0.4%). Per publication, this prevalence amounted to 5.5% (± 0.3%) of the publications with a bar chart. In our prediction pipeline, we predict that there are 479 proportional ink violations of the 20,666 bar charts that could be processed (2.3% (± 0.1%) per bar chart, 3.6% (± 0.2%) per publication with bar chart).*”

Comment 3: How do you deal with the fact that bar charts are used very differently in different disciplines? How is that reflected in your statistics?

Response: Some research fields are less likely to use bar charts in their publications. For section 3.2.4, we computed the mean of the likelihood of having a violation of the principle of proportional ink for each field in Figure 4. In another word, the likelihood in section 3.2.4 is normalized.

Comment 4: After reading the article, it is still not clear to me whether the graphical integrity classification system developed by you allows a sufficient statement about the integrity of a graph, or whether there are — similar to other types of images, or even texts — gray areas that have to be included when deciding about the integrity. So, at what point is a bar chart to be labeled "inappropriate" — how many criteria have to be considered to make a reliable judgment? Possibly some concrete examples could provide clarity here and strengthen your argumentation?

Response: Thanks for your comment. The reviewer is absolutely correct in that there are various reasons why we are finding apparent violations of graphical principles. We certainly do not attach intentionality to

our findings and also believe that there are degrees of intentionality, from a simple mistake to a purposely misleading attempt. We would invite the reviewers to see our response to comment #5 of reviewer #1 and comment #5 from reviewer #2. In particular, “inappropriate” bar charts violate the principle of proportional ink. Such violations are the following:

“ *The specific rules are: a bar chart's y-axis should start from zero, have one scale, and not be partially hidden (Bergstrom & West, 2020; Tufte, 2001).*” (in section 4.1.1) These are our criteria for our annotations of violations of principle of proportional ink in bar charts of our sample.

For our automated classification system, we transformed these mentioned criteria as features for our classifier (see Table 3, in the main text). Our features are extracted information in images, through from our feature engineering. We added some examples in Figure 3 (in supplementary material) to show our method can extract relatively accurate information for our feature engineering.Then we use machine learning models to learn the subtle differences between “appropriate” bar charts and “inappropriate” bar charts. Our machine learning model thus found a relationship between features and “inappropriate” bar charts after training on human annotated images.

Comment 5: in section 3.2.4, you note that there are significant differences between academic disciplines when it comes to graphical integrity. Have you explored whether these differences may also be because there are guidelines for handling images and graphs in certain disciplines and in others not?

Response: This is an important question. We reviewed graphic design literature, and we found graphic design papers or books are general and do not write to specific disciplines. However, in terms of research integrity, researchers from different disciplines do perceive different levels of regulations (Haven et al., 2019). Thus, it is possible that some disciplines have more attention on the quality of graphs.

We have added the following to section 5. Discussion: “*Our country and journal differences differ from image integrity studies (e.g., (Bik et al., 2016)). For example, we found that European countries tend to have a higher likelihood of graphical integrity violations. The difference in proportional ink violation across fields might be the result of the coverage of research regulation across research fields. For example, some fields have better research integrity regulations than others (Haven et al., 2019).*”

Comment 6: in section 3.2.5, it remains unclear what criteria you used exactly to undertake the investigation "by country". When you compare „countries“, does that mean you compare the publications of journals published in a given country or is it about the origin of the authors? Or something completely different?

Response: Thanks for asking us to clarify this important point. We used authors' affiliation countries for the analysis. We have added a clarification to section 3.2.5 to resolve this confusion:

“*Here, we examined the likelihood of having articles with graphical integrity issues for all their authors from the country of their affiliations.*”

Comment 7: Subject-specific abbreviations should be explained to facilitate understanding (e.g. p. 5, item 3.1.2 ROC / AUC etc.).

Response: Across reviewers, a common point was that our evaluation needed more discussion. We now explain the ROC and AUC abbreviations in section 3.1.2 and significantly expand our evaluation explanation in section 4.2.3. We added the following to section 3.1.2 (pp. 6):

"*We used Area under the ROC curve (denoted as AUC), precision, and recall with stratified 5-fold cross-validation to evaluate the performance of our graphical integrity evaluator (for our evaluation protocol, see Materials and Methods section 4.2.3). AUC indicates the ability of our detector to classify two classes, and our average AUC across folds is 0.917 with a standard deviation of 0.02.*"

Comment 8: Under Data and Code Availability it is implied that all data and code will be published via GitHub and Zenodo. However, I could not find a corresponding link.

Response: The reviewer is correct that we did not include this in our manuscript as we were expecting to do it after the manuscript was accepted. We apologize. We have created a code repository and a data repository. The link to our code repository: https://github.com/sciosci/graph_check. The link to our data repository: https://zenodo.org/record/5500684#.YTvYclspBhF. They will be both public upon publication.

Reference

Armond, A. C. V., Gordijn, B., Lewis, J., Hosseini, M., Bodnár, J. K., Holm, S., & Kakuk, P. (2021). A scoping review of the literature featuring research ethics and research integrity cases. *BMC Medical Ethics*, *22*(1), 50. https://doi.org/10.1186/s12910-021-00620-8

Beattie, V., & Jones, M. J. (2002). Measurement distortion of graphs in corporate reports: An experimental study. *Accounting, Auditing & Accountability Journal*, *15*(4), 546–564. https://doi.org/10.1108/09513570210440595

Bergstrom, C. T., & West, J. D. (2020). *Calling Bullshit: The Art of Skepticism in a Data-Driven World* (Illustrated Edition). Random House.

Bertin, J. (2011). *Semiology of Graphics: Diagrams, Networks, Maps*. ESRI Press.

DarrinEide. (2021). *FAQ about Microsoft Academic Graph—Microsoft Academic Services*. https://docs.microsoft.com/en-us/academic-services/graph/resources-faq

Desmond, H., & Dierickx, K. (2021). Research integrity codes of conduct in Europe: Understanding the divergences. *Bioethics*, *35*(5), 414–428. https://doi.org/10.1111/bioe.12851

Haven, T. L., Tijdink, J. K., Martinson, B. C., & Bouter, L. M. (2019). Perceptions of research integrity

climate differ between academic ranks and disciplinary fields: Results from a survey among academic researchers in Amsterdam. *PLOS ONE*, *14*(1), e0210599. https://doi.org/10.1371/journal.pone.0210599

Hutson, M. (2018). Artificial intelligence faces reproducibility crisis. *Science*, *359*(6377), 725–726. https://doi.org/10.1126/science.359.6377.725

Swan, A., & Brown, S. (2004). Authors and open access publishing. *Learned Publishing*, *17*(3), 219–224. https://doi.org/10.1087/095315104323159649

Tufte, E. R. (2001). *The visual display of quantitative information* (Vol. 2). Graphics press Cheshire, CT.

Ware, C. (2010). *Visual Thinking for Design: For Design*. Elsevier.
