# Supplementary material for: Graphical integrity issues in open access publications: Detection and patterns of proportional ink violations
Source: PLoS Comput Biol. 2021 Dec 13;17(12):e1009650. doi: 10.1371/journal.pcbi.1009650 (PMC8700024; doi:10.1371/journal.pcbi.1009650)
Supplement: Attachment — Submitted filename: zhuang_huang_acuna_revision3_response.pdf [file pcbi.1009650.s011.pdf]

“Graphical integrity issues in open access publications: detection and patterns of proportional ink violations”: response to reviewers round 2

Han Zhuang, Tzu-Yang Huang, and Daniel E. Acuna
School of Information Studies
Syracuse University

Thanks to the editor for managing a smooth review process. We also thank the reviewers for their words. We would not have been able to polish our manuscript to this degree without their help.

We have fixed all typographical errors. We honestly do not know how those slipped through our internal reading, but thank you for listing them.

Regarding reviewer #3 comment that we seem to suggest that “the problem of ink violations is relatively harmless, but some paragraphs later in your conclusions, you make the point that the misunderstanding of a graph could well harm peoples lives.” We have revised this point because we *do* believe this issue is problematic and potentially harmful. Still, we are not sure whether releasing the tool is the right approach to combat such issues. We are releasing the code per the journal’s guidelines and for good open science practice, but the installation and usage take some time and effort. Hopefully, this investment is a natural barrier for bad actors. We think the proper cost-benefit analysis of the situation would imply that releasing the tool in a limited fashion is better overall.
